# Synthesis and Thermal Properties of Bio-Based Janus Ring Siloxanes Incorporating Terpenes and Terpenoids

**DOI:** 10.3390/ma17215348

**Published:** 2024-10-31

**Authors:** Niyaz Yagafarov, Jiaorong Kuang, Nobuhiro Takeda, Yujia Liu, Armelle Ouali, Masafumi Unno

**Affiliations:** 1 Department of Chemistry and Chemical Biology, Gunma University, 1-5-1 Tenjin-cho, Kiryu 376-8515, Japan; driagafarov@gunma-u.ac.jp (N.Y.); t221a036@gunma-u.ac.jp (J.K.); ntakeda@gunma-u.ac.jp (N.T.); unno@gunma-u.ac.jp (M.U.); 2 ICGM, Univ Montpellier, CNRS, ENSCM (Institut Charles Gerhardt Montpellier, Université de Montpellier, Centre National de la Recherche Scientifique, Ecole Nationale Supérieure de Chimie de Montpellier), 1919 Route de Mende, CEDEX 05, 34293 Montpellier, France

**Keywords:** Janus ring siloxane, silsesquioxane, hydrosilylation, terpenes, terpenoids, all-*cis*-cyclotetrasiloxane

## Abstract

A mild and highly selective hydrosilylation method was employed to synthesize five novel well-defined Janus ring siloxanes bearing terpenes and terpenoids, which are the main bioactive components of essential oils. The characterization of these new bio-sourced molecular materials, derived from hydrosilyl-substituted all-*cis*-cyclotetrasiloxane, was conducted through comprehensive analyses using multinuclear NMR, infrared spectroscopy, elemental analysis, and mass spectroscopy. The thermal stability of the newly synthesized Janus rings was investigated, and the siloxane skeleton was shown to confer an enhanced thermal stability compared with free terpenes and terpenoids.

## 1. Introduction

Silsesquioxanes (SQ), organosilicon compounds with each silicon atom bonded to three oxygen atoms and one carbon substituent, exhibit good biocompatibility, excellent heat resistance, and mechanical strength owing to their robust silicon–oxygen network. Additionally, the presence of organic substituents enhances their flexibility and functional extensibility, making them attractive organic–inorganic hybrid materials for various applications, including functional polymers, catalysis, and biomedicine [1,2,3,4,5,6,7].

**Scheme 1:**
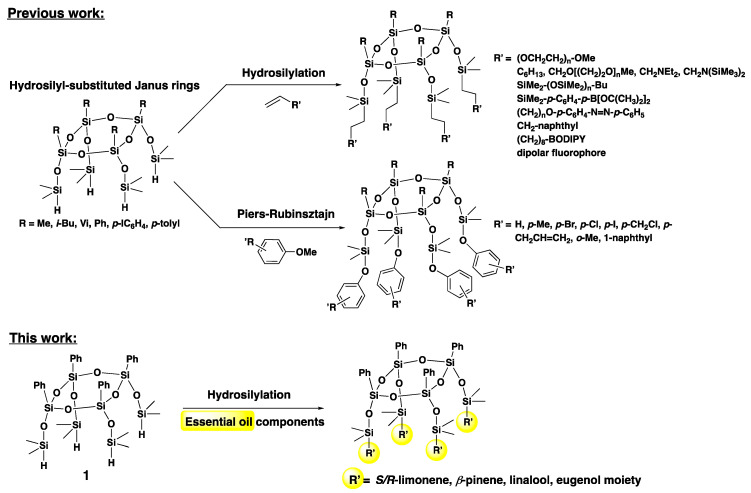
Functionalization of hydrosilyl-substituted Janus rings has been reported in previous studies and in this work.

All-*cis*-cyclotetrasiloxanetetraol derivatives (all-*cis*-T_4_) serve as high-efficiency precursors for the preparation of well-defined molecular SQs. The synthesis and functionalization of these all-*cis*-T_4_ compounds have been intensively studied owing to their Janus-type structure, featuring two different functional groups on the upper and lower faces, resulting in their designation as “Janus rings” [8]. Various reactive groups such as hydrosilyl, alkenyl, hydroxy, and halogen-substituted Janus rings have been successfully synthesized with good yields. The organic chains on the upper and lower faces of these compounds can be extended through reactive substituents using common organic transformations [8]. Hydrosilyl-substituted all-*cis*-T_4_ is the most commonly used precursor for fabricating T_4_-based hybrid materials owing to the high reactivity of the Si-H group [9,10,11,12,13]. The latter can be readily functionalized via platinum-catalyzed hydrosilylation with olefins. This strategy has been employed to introduce a wide range of hydrophilic/hydrophobic fragments [14,15], oligo/polymer siloxane chains [16,17,18,19], organoboron groups [20], azobenzene [21], naphthalene [22], BODIPY (boron-dipyrromethene) dyes [23,24], and dipolar fluorophores into all-*cis*-T_4_ structures (Scheme 1) [25]. The Piers–Rubinsztajn reaction is another convenient synthetic method to functionalize Si-H bonds. For example, the preparation of well-controlled siloxane materials consisting of all-*cis*-T_4_-bearing anisole derivatives has been reported (Scheme 1) [26,27,28]. Regardless of the synthetic method, T_4_-based hybrid building blocks find applications in the preparation of coatings [16], photoactive/fluorescent materials [21,22,23,24,25], and sensors [28].

Terpenes and terpenoids, which are the main components of essential oils, are renewable organic compounds and highly important bioactive molecules [29]. Essential oils are volatile oils obtained from plants through distillation, expression, or solvent extraction. Not only are essential oils used in cosmetics, perfumes, and flavoring, but they also exhibit various biological activities, such as antimicrobial, antioxidant, anti-inflammatory, antitumor, allelopathic, repellent, antifungal, and insecticidal activities [30,31,32]. This versatility makes their terpene and terpenoid components (e.g., limonene, eugenol, linalool, and pinene) potentially valuable in the pharmaceutical, therapeutic, agrochemical, and food preservative fields [33,34]. In addition to their bioactivities, terpenes and terpenoids are attractive building blocks for the preparation of bio-based materials. Hence, they have been incorporated into various materials, including bioplastics, biopolymers [35], and siloxane-based materials [36,37,38,39,40,41,42,43,44,45,46]. The latter are prepared through the hydrosilylation of the terpene alkene function with the Si-H bonds present in the siloxane precursor. For example, spherosilicates containing eight limonene fragments can serve as additives to polylactide, which is used in 3D printing, and the resulting material exhibits improved processing properties, including adhesion, rheology, and mechanical properties [41]. Similarly, eugenol-functionalized siloxane-based materials showed superior thermal stability, mechanical properties, and hydrophobicity compared to those lacking eugenol [36,38,40,42,43,44]. They can be used as anticorrosion additives in epoxy resins and hybrid polyurethanes [36,43], LED (light-emitting diode) encapsulants [42], and silane coupling agents [44]. Eugenol and linalool were also grafted onto linear polysiloxane and triethoxysilane, and the resulting materials were employed in the preparation of coatings with high antiadhesive properties [46]. In the field of biomedicine, eugenol-grafted MQ silicone resins (silicone resins built of M- and Q-unit siloxanes) exhibit antibacterial properties [40].

Interestingly, despite extensive research on the functionalization of all-*cis*-T_4_ and the crucial role of terpenes and terpenoids as building blocks to obtain bio-based siloxane materials, the incorporation of essential oil components into all-*cis*-T_4_ has not yet been reported. In this study, a series of all-*cis*-cyclotetrasiloxanes was synthesized through selective hydrosilylation, starting from Si-H-substituted all-*cis*-tetraphenylcyclotetrasiloxane (**1**) and S/R-limonene, (-)-*β*-pinene, linalool, and eugenol (Scheme 1). The prepared materials were thoroughly characterized and exhibited an improved thermal stability compared with molecules lacking a siloxane core. This research opens new possibilities for the use of well-defined cyclic siloxanes in the development of new functional hybrid materials.

## 2. Materials and Methods

### 2.1. General Consideration

All the reactions were performed within an argon atmosphere using the standard Schlenk technique. Tetrahydrofuran (THF) and toluene were dried using an mBRAUN purification system. Triethylamine (Et_3_N) was distilled from potassium hydroxide and stored in potassium hydroxide within an argon atmosphere. Karstedt’s catalyst (in xylene, 2% Pt) was purchased from Sigma-Aldrich, Tokyo, Japan, while (*S*)-(−)-limonene and (*R*)-(+)-limonene were purchased from Kanto Chemical Co. Inc., Tokyo, Japan; (-)-*β*-pinene, linalool, and eugenol were purchased from TCI Co., Ltd., Tokyo, Japan, and all reagents were used as received without further purification. The Fourier transformation nuclear magnetic resonance (NMR) spectra were obtained using a JEOL JNM-ECA 600 (^1^H at 600.17 MHz, ^13^C at 150.91 MHz, ^29^Si at 119.24 MHz) NMR instrument (JEOL Ltd., Akishima, Tokyo, Japan). MALDI-TOF (matrix-assisted laser desorption/ionization coupled time-of-flight) mass analysis was carried out with a Shimadzu AXIMA^®^ instrument (Shimadzu Corporation, Kyoto, Japan). Infrared (IR) spectra were obtained using a Shimadzu IR Spirit FTIR spectrometer (Shimadzu Corporation, Kyoto, Japan). Thermal gravimetric analysis (TGA) was carried out using a Rigaku (Tokyo, Japan) under nitrogen flow (250 mL min^−1^) at a heating rate of 10 °C min^−1^. The samples with siloxane cores were measured at temperatures ranging from 50 to 1000 °C, where they remained for 5 min. Essential oil components were measured at temperatures ranging from 30 to 300 °C, where they remained for 5 min. The weight loss and heating rate were continuously recorded during the experiment. Optical rotations were measured using an automatic polarimeter (AP-300, ATAGO, Tokyo, Japan).

### 2.2. Synthesis of Compound ***1*** Starting from All-Cis Tetraphenylcyclotetrasiloxanetetraol

An argon-purged, three-necked, round bottom flask equipped with a stir bar was charged with all-*cis* cyclic silanol (1.0 g, 1.8 mmol), dry THF (100 mL) and distilled triethylamine (1.5 mL, 10.8 mmol). The mixture was cooled to 0 °C for 30 min and chlorodimethylsilane (1.2 mL, 10.8 mmol) was added dropwise into the mixture under argon at −5 °C by hand. When the addition was complete, the mixture was stirred at −5 °C for 1 h, warmed to room temperature, and stirred for 23 h at room temperature. Water (150 mL) was added to the reaction mixture, which was then extracted 3 times with 50 mL of hexane. The gathered organic layer was washed three times with brine, dried over anhydrous sodium sulfate (Na_2_SO_4_), evaporated on a rotavapor, and dried under vacuum at room temperature for one day to afford pure compound **1** [26] (1.13 g, 80%) as a white solid that could be used without purification.

### 2.3. Synthesis of Compounds ***2***–***6***

Synthesis of Compound **2**. An argon-purged, two-necked, round bottom flask equipped with a stir bar was charged with compound **1** (0.05 g, 0.064 mmol), S-limonene (47 μL, 0.29 mmol), and dry toluene (0.3 mL). Karstedt’s catalyst (2% Pt, commercial bottle, 7.3 μL, 0.6 μmol) was added into the mixture under argon atmosphere at room temperature. After the addition, the mixture was stirred at 40 °C for 24 h. After the reaction, the mixture was passed through a silica plug with diethyl ether. The crude product was purified by column chromatography (silica gel, eluent: dichloromethane (CH_2_Cl_2_)) to afford the desired product as a colorless oil (0.064 g, 75%).

Analysis data: ^1^H NMR (600.17 MHz, CDCl_3_): δ = 0.21 (s, 12H), 0.22 (s, 6H), 0.22 (s, 6H), 0.45–0.54 (m, 4H), 0.72–0.79 (m, 4H), 0.88 (d, *J* = 6.9 Hz, 12H), 1.13–1.31 (m, 8H), 1.53–1.74 (m, 22H), 1.86–1.91 (m, 12H), 5.35–5.36 (m, 4H), 7.03–7.07 (m, 8H), 7.23–7.28 (m, 12H) ppm. ^13^C{^1^H} NMR (150.91 MHz, CDCl_3_): δ = 1.38, 1.56, 1.63, 19.17, 19.49, 22.98, 23.34, 23.63, 25.50, 26.82, 27.97, 28.93, 31.09, 31.16, 32.93, 33.11, 41.26, 41.34, 121.25, 121.28, 127.41, 129.72, 133.35, 133.97, 133.99, 134.17 ppm. ^29^Si{^1^H} NMR (119.24 MHz, CDCl_3_): δ = −79.87, 11.06, 11.12 ppm. MALDI-TOF MS (m/z): 1353.59 ([M + Na]^+^, calcd 1353.64); 1369.56 ([M + K]^+^, calcd 1369.62). Elemental analysis: Calcd for C_72_H_112_O_8_Si_8_: C, 65.00; H, 8.49; Found: C, 63.90; H, 8.41. [α]_D_^25^ = −31.38° (*c* = 1, EtOAc).

Synthesis of Compound **3**. An argon-purged, two-necked, round bottom flask equipped with a stir bar was charged with compound **1** (0.1 g, 0.13 mmol), R-limonene (95 μL, 0.59 mmol), and dry toluene (0.6 mL). Karstedt’s catalyst (2% Pt, commercial bottle, 15.0 μL, 1.3 μmol) was added to the mixture under an argon atmosphere at room temperature. After the addition, the mixture was stirred at 40 °C for 24 h. After the reaction, the mixture was passed through a silica plug containing diethyl ether. The crude product was purified by column chromatography (silica gel, eluent: CH_2_Cl_2_) to afford the desired product, a colorless oil (0.13 g, 77%).

Analysis data: ^1^H NMR (600.17 MHz, CDCl_3_): δ = 0.20 (s, 12H), 0.21 (s, 6H), 0.21 (s, 6H), 0.44–0.53 (m, 4H), 0.71–0.77 (m, 4H), 0.86 (d, *J* = 6.9 Hz, 12H), 1.11–1.32 (m, 8H), 1.56–1.65 (m, 22H), 1.85–1.90 (m, 12H), 5.32–5.36 (m, 4H), 7.02–7.06 (m, 8H), 7.22–7.26 (m, 12H) ppm. ^13^C{^1^H} NMR (150.91 MHz, CDCl_3_): δ = 1.39, 1.57, 1.63, 19.17, 19.49, 22.99, 23.35, 23.63, 25.50, 26.83, 27.97, 28.93, 31.09, 31.16, 32.93, 33.12, 41.26, 41.35, 121.25, 121.29, 127,41, 129.72, 133.35, 133.97, 133.99, 134.18 ppm. ^29^Si{^1^H} NMR (119.24 MHz, CDCl_3_): δ = −79.87, 11.04, 11.10 ppm. MALDI-TOF MS (*m*/*z*): 1353.66 ([M + Na]^+^, calcd 1353.64); 1369.65 ([M + K]^+^, calcd 1369.62). Elemental analysis: Calcd for C_72_H_112_O_8_Si_8_: C, 65.00; H, 8.49; Found: C, 62.66; H, 8.10. [α]_D_^25^ = +32.40° (c = 1, EtOAc).

Synthesis of Compound **4**. An argon-purged, two-necked, round bottom flask equipped with a stir bar was charged with compound **1** (0.1 g, 0.13 mmol), (-)-*β*-pinene (92 μL, 0.59 mmol), and dry toluene (0.6 mL). Karstedt’s catalyst (2% Pt, commercial bottle, 15.0 μL, 1.3 μmol) was added to the mixture under an argon atmosphere at room temperature. After the addition, the mixture was stirred at 40 °C for 24 h. After the reaction, the mixture was passed through a silica plug containing diethyl ether. The crude product was purified by column chromatography (silica gel, eluent: CH_2_Cl_2_) to afford the desired product, a colorless oil (0.11 g, 65%).

Analysis data: ^1^H NMR (600.17 MHz, CDCl_3_): δ = 0.21 (s, 24H), 0.56–0.69 (m, 8H), 0.74 (s, 12H), 1.13 (s, 12H), 1.18–1.32 (m, 8H), 1.56–1.67 (m, 8H), 1.78–1.82 (m, 4H), 1.93–1.98 (m, 4H), 2.08–2.13 (m, 4H) 7.04–7.08 (m, 8H), 7.22–7.28 (m, 12H) ppm. ^13^C{^1^H} NMR (150.91 MHz, CDCl_3_): δ = 1.63, 1.87, 20.14, 23.14, 24.91, 25.34, 26.42, 27.04, 30.55, 39.60, 40.76, 49.34, 127.42, 129.70, 133.93, 134.18 ppm. ^29^Si{^1^H} NMR (119.24 MHz, CDCl_3_): δ = −79.89, 10.66 ppm. MALDI-TOF MS (m/z): 1352.43 ([M + Na]^+^, calcd. 1352.60); 1368.46 ([M + K]^+^, calcd. 1368.60). Elemental analysis: Calcd for C_72_H_112_O_8_Si_8_: C, 65.00; H, 8.49; Found: C, 63.09; H, 8.21.

Synthesis of Compound **5**. An argon-purged, two-necked, round bottom flask equipped with a stir bar was charged with compound **1** (0.1 g, 0.13 mmol), linalool (105 μL, 0.59 mmol), and dry toluene (0.6 mL). Karstedt’s catalyst (2% Pt, commercial bottle, 15.0 μL, 1.3 μmol) was added to the mixture under an argon atmosphere at room temperature. After the addition, the mixture was stirred at 40 °C for 24 h. After the reaction, the mixture was passed through a silica plug containing diethyl ether. The crude product was purified by column chromatography (silica gel, eluent: CH_2_Cl_2_) to afford the desired product, a colorless viscous oil (0.12 g, 70%).

Analysis data: ^1^H NMR (600.17 MHz, CDCl_3_): δ = 0.23 (s, 24H), 0.56–0.60 (m, 8H), 1.06 (s, 12H), 1.37–1.41 (m, 16H), 1.44 (brs, 4H), 1.60 (s, 12H), 1.69 (s, 12H), 1.93–1.97 (m, 8H), 5.08–5.11 (m, 4H), 7.07–7.11 (m, 8H), 7.25–7.31 (m, 12H) ppm. ^13^C{^1^H} NMR (150.91 MHz, CDCl_3_): δ = 0.30, 11.67, 17.75, 22.72, 25.83, 26.05, 35.15, 40.97, 73.13, 124.75, 127.55, 129.96, 131.52, 133.01, 134.08 ppm. ^29^Si{^1^H} NMR (119.24 MHz, CDCl_3_): δ = −79.36, 11.72 ppm. MALDI-TOF MS (m/z): 1424.59 ([M + Na]^+^, calcd. 1424.70); 1440.59 ([M + K]^+^, calcd. 1440.70). Elemental analysis: Calcd for C_72_H_120_O_12_Si_8_: C, 61.66; H, 8.63; Found: C, 60.54; H, 8.54.

Synthesis of Compound **6**. An argon-purged, two-necked, round bottom flask equipped with a stir bar was charged with compound **1** (0.1 g, 0.13 mmol), eugenol (91 μL, 0.59 mmol), and dry toluene (0.6 mL). Karstedt’s catalyst (2% Pt, commercial bottle, 15.0 μL, 1.3 μmol) was added to the mixture under an argon atmosphere at room temperature. After the addition, the mixture was stirred at 40 °C for 24 h. After the reaction, the mixture was passed through a silica plug containing diethyl ether. The crude product was purified by column chromatography (silica gel, eluent: CH_2_Cl_2_) to afford the desired product, a colorless viscous oil (0.1 g, 53%).

Analysis data: ^1^H NMR (600.17 MHz, CDCl_3_): δ = 0.21 (s, 24H), 0.65–0.69 (m, 8H), 1.57–1.66 (m, 8H), 2.51 (t, *J* = 7.7 Hz, 8H), 3.80 (s, 12H), 5.53 (s, 4H), 6.61–6.62 (m, 8H), 6.81–6.83 (m, 4H), 7.07–7.11 (m, 8H), 7.27–7.32 (m, 12H) ppm. ^13^C{^1^H} NMR (150.91 MHz, CDCl_3_): δ = 0.44, 18.03, 25.63, 39.47, 55.89, 111.10, 114.20, 121.07, 127.49, 129.86, 133.09, 134.06, 134.65, 143.63, 146.34 ppm. ^29^Si{^1^H} NMR (119.24 MHz, CDCl_3_): δ = −79.54, 10.79 ppm. MALDI-TOF MS (m/z): 1464.31 ([M + Na]^+^, calcd. 1464.50); 1480.32 ([M + K]^+^, calcd. 1480.50). Elemental analysis: Calcd for C_72_H_96_O_16_Si_8_: C, 59.96; H, 6.71; Found: C, 59.22; H, 6.28.

## 3. Results and Discussion

**Scheme 2:**
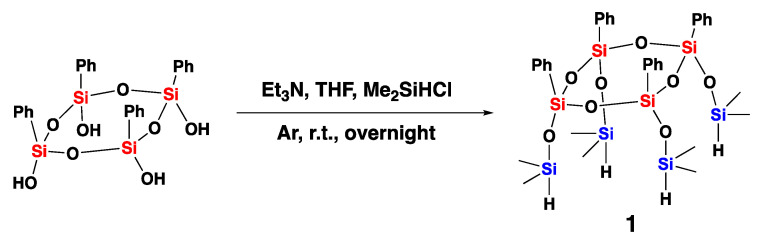
Synthesis of compound **1**; red Si: T-unit Si; blue Si: M-unit Si.

Hydrosilyl-substituted well-defined all-*cis*-cyclotetrasiloxane (**1**) was selected as the precursor for this study because of its accessibility and facile preparation from commercially available and cheap chlorodimethylsilane and large-scale synthesizable all-*cis*-tetraphenylcyclotetrasiloxane tetraol [26]. Additionally, the Si-H groups in all-*cis*-cyclotetrasiloxane (**1**) exhibit a high reactivity towards hydrosilylation reactions with various olefins [47]. Compound **1** was initially allowed to react with optically active *S*-limonene in dry toluene at room temperature for one day in the presence of 1 mol% Pt per Si-H bond (Scheme 2). After the reaction, the catalyst was removed by using a silica plug. According to the proton NMR spectrum of the obtained crude product, the signals around 4.87 ppm assigned to the Si-H substituents [26] did not completely disappear, indicating that the reaction was incomplete, with approximately 80% of the Si-H substituents being converted, and di-and tri-substituted byproducts were formed. Consequently, the reaction temperature was increased to 40 °C, and the reaction was allowed to proceed for one day in the presence of 0.25 mol% Pt per Si-H group. To our delight, the reaction was then complete, as evidenced by the total disappearance of the Si-H group signals in the ^1^H NMR spectrum. The optimized reaction conditions required a comparable amount of Pt catalyst and a lower temperature than those reported in most studies related to siloxane-based materials [37,38,39,40,41,43]. The crude product was purified by silica gel column chromatography to remove the slight excess of *S*-limonene, yielding pure product **2** as a colorless oil in a 75% yield.

**Scheme 3:**
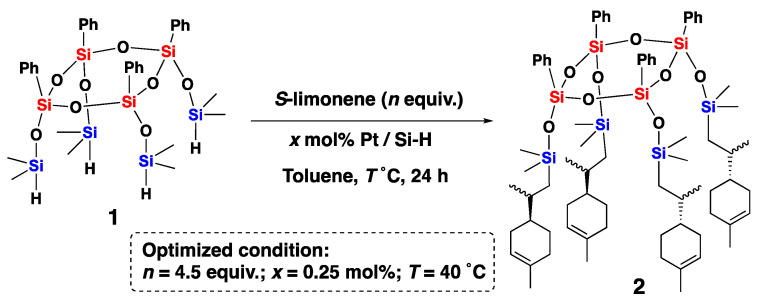
Hydrosilylation of **1** with *S*-limonene; red Si: T-unit Si; blue Si: M-unit Si.

**Scheme 4:**
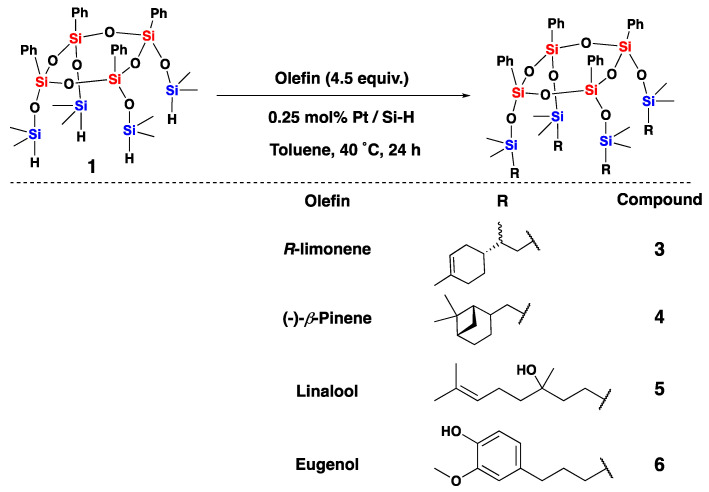
Hydrosilylation of **1** with different essential oil components; red Si: T-unit Si; blue Si: M-unit Si.

Under the optimized reaction conditions (Scheme 3), compound **1** was reacted with a widely available and low-cost stereospecific pure *R*-limonene (Scheme 4) [48]. After purification by column chromatography, compound **3** was obtained as a colorless oil with a good yield of 77%.

**Figure 1:**
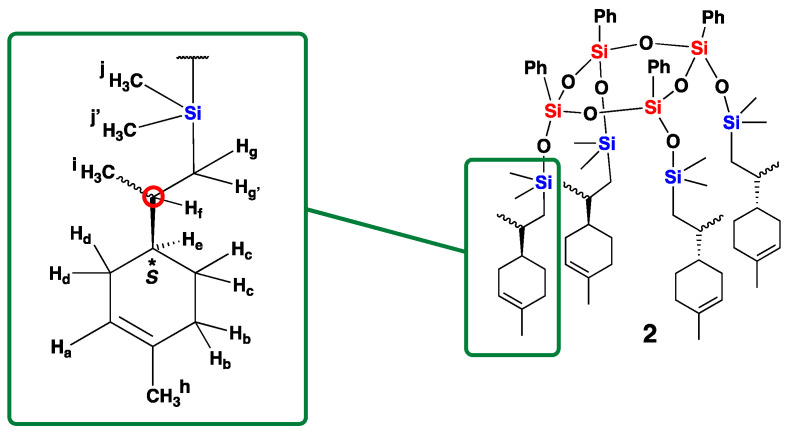
Zoomed-in view of the incorporated *S*-limonene moiety in compound **2**; red Si: T-unit Si; blue Si: M-unit Si; red circle: an asymmetric carbon atom.

The ^1^H and ^29^Si NMR analysis results for compounds **2** and **3** were quite similar (see Supplementary Materials, Figures S1 and S4). In the ^29^Si NMR analyses of compounds **2** and **3**, two distinct signal patterns were observed in both cases, one signal at −79.87 ppm corresponded to the T-unit silicon atoms, and two nearby signals at 11.06 and 11.12 ppm for compound **2**, and at 11.04 and 11.10 ppm for compound **3**, corresponded to the M-unit silicon atoms (see Table 1, Supporting Information, Figures S3 and S6). This indicated the existence of two different types of M-unit silicon atoms for compounds **2** and **3**, confirming that the four extended siloxane chains on the same face are not chemically equivalent. On the ^1^H NMR spectrum of compound **2**, the methylene groups (Figure 1, H_g_, H_g’_) adjacent to the M-unit silicon atoms resulting from the hydrosilylated double bonds of isoprene showed two multiplets at 0.72–0.79 ppm and 0.45–0.54 ppm, respectively. This demonstrates that hydrosilylation occurred successfully and that the two protons on the methylene group were diastereotopic and, hence, not magnetically equivalent. This inequivalence is due to the presence of an adjacent chiral carbon (Figure 1, carbon in the red circle). The signal of the olefinic protons of the cyclohexenyl group (Figure 1, H_a_) was clearly observed at 5.4 ppm, and signals attributable to methyl groups (Figure 1, H_i_) were observed at 0.88 ppm as a doublet, indicating that the hydrosilylation selectively occurred only on the isoprene groups of *S*-limonene.

The CH_3_ (H_h_), CH_2_ (H_b_, H_c_, H_d_), and CH (H_e_) groups of cyclohexenes and CH_f_ were observed in the range of 1.13–1.91 ppm (Figure 1). The peaks corresponding to the phenyl groups on the T-unit silicon atoms (silicon atoms connected to three oxygen atoms) exhibited two distinct patterns at 7.03–7.07 ppm and 7.23–7.28 ppm. The ^13^C NMR spectra were in accordance with the structures of compounds **2** and **3** (see Supplementary Materials, Figures S2 and S5).

The structures of these two compounds were verified using MALDI-TOF mass spectrometry (see Supplementary Materials, Figures S16 and S17), with experimental ([M + Na]^+^ found at 1353.59 g mol^−1^ for **2** and 1353.66 g mol^−1^ for **3,** aligning well with the calculated value of 1353.64 g mol^−1^.

To broaden the scope of substrates derived from essential oils, compound **1** was reacted with monoterpene and phenol derivatives (Scheme 4). Therefore, another bicyclic terpene, (-)-*β*-pinene, was utilized under the above optimized conditions, leading to the isolation of target compound **4** as a colorless viscous oil in 65% yield after filtration through a silica plug and purification via column chromatography. The ^1^H NMR analysis of **4** revealed the complete disappearance of the Si-H groups from precursor **1** and the double bond from (-)-*β*-pinene (see Supplementary Materials, Figure S7). The Si-C*H*_2_ formed after hydrosilylation provided signals in the range of 0.56–0.69 ppm, and the methyl groups on the M-unit silicon atoms displayed a singlet at 0.21 ppm. Additionally, the two methyl groups on the pinene moieties were observed as singlets at 0.74 ppm and 1.13 ppm, respectively. The other C*H*_2_ and C*H* groups of the pinene moieties showed signals between 1.18 and 2.13 ppm.

Subsequently, the scope was extended to include an unsaturated monoterpene alcohol, linalool, which underwent hydrosilylation with compound **1**, resulting in the isolation of the corresponding product (**5**) as a colorless viscous oil in 70% yield. In the proton NMR spectrum of **5**, a multiplet signal assigned to the proton of (CH_3_)_2_C=C*H* was displayed at 5.08–5.11 ppm (Supplementary Materials, Figure S10). The methylene groups (Si-C*H*_2_ and Si-CH_2_-C*H*_2_) formed after the hydrosilylation of the terminal double bond (H_2_C=CH-) afforded two signals at 0.56–0.60 ppm and 1.37–1.42 ppm, respectively, with the latter ones overlapping with the peaks for the methylene groups and OH groups. This demonstrates the high selectivity of hydrosilylation towards less-bulky alkenyl groups. The methyl groups on the M-unit silicon atoms, on the carbon adjacent to the OH groups, and on the alkenyl groups exhibited four distinctive singlets at 0.23 ppm, 1.06 ppm, 1.60 ppm and 1.69 ppm, respectively.

The synthesis of siloxane materials using eugenol has also been attempted. To our delight, the hydrosilylation of eugenol with **1** was completed, and the desired product (**6**) was obtained as a colorless viscous oil in 53% isolated yield. The ^1^H NMR spectrum showed a total conversion of allyl groups, and three pattern signals corresponding to three methylene groups between the M-unit silicon atoms and C_Ar_ were observed at 0.65–0.69 ppm, 1.57–1.66 ppm, and 2.51 ppm. The peaks for the hydroxy and methoxy groups connected to the aromatic rings were located at 5.53 ppm and 3.80 ppm as singlets, respectively. The aromatic protons were observed in the range of 6.61–7.32 ppm (see Supplementary Materials, Figure S13).

The ^29^Si NMR analysis for compounds **4**, **5**, and **6** displayed two distinctive single peaks for T-unit and M-unit silicon atoms, respectively, at −79.89 ppm and 10.66 ppm for compound **4**, −79.36 ppm and 11.72 ppm for compound **5**, and −79.54 ppm and 10.79 ppm for compound **6**, indicating the well-defined symmetrical structures of these compounds (see Table 1, Supplementary Materials, Figures S9, S12 and S15). ^13^C NMR analysis, MALDI-TOF mass spectroscopy (see Supplementary Materials, Figures S8, S11, S14 and S18–S20) and elemental analysis confirmed the structures and purity of compounds **4**–**6** as well.

**Figure 2:**
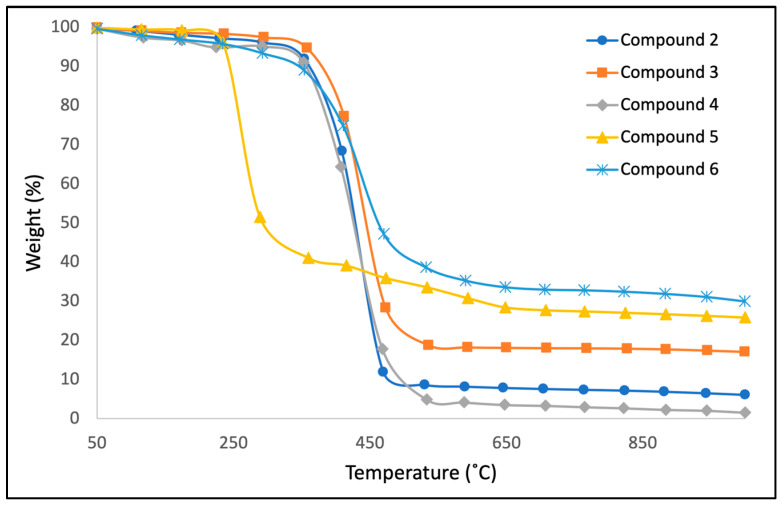
TGA graphs of compounds **2**–**6**.

The thermal properties of the bio-based molecular materials (**2**–**6**) were compared to those of free terpenes and terpenoids using thermogravimetric analysis (TGA). Overall, the latter exhibited a low 5% weight loss temperature (Td_5_). Specifically, eugenol possessed a Td_5_ equal to 107 °C, whereas *S*/*R*-limonene, (-)-*β*-pinene, and linalool showed a Td_5_ lower than 100 °C. For all of these molecules, at 300 °C, less than 20% of the initial weight remained, and eugenol completely evaporated at 167 °C (see Supplementary Materials, Figure S26). In comparison, the compounds with a T_4_ cyclic siloxane core (**2**–**6**) showed a significant increase in Td_5_, with Td_5_ values equal to 322 °C for **2**, 354 °C for **3**, and 296 °C for **4**, an increase of approximately 250 °C compared to those of free *S*/*R*-limonene and (-)-*β*-pinene, and Td_5_ values equal to 239 °C for **5**, 256 °C for **6**, an increase of approximately 150 °C compared to free linalool and eugenol. At 300 °C, more than 40% of the initial weight of compounds **2**–**6** remained (Figure 2), in contrast to less than 20% for the free terpenes and terpenoids.

The observed thermal properties of compounds **2**–**6** indicate that the incorporation of the cyclic siloxane skeleton enhanced the thermal stability of terpenes and terpenoids. Notably, the Td_5_ values of compounds **2**–**6** were all higher than those of compound **1** (228 °C) under identical conditions. This enhancement can be attributed to the synergistic effect between the siloxane core and the terpene substituents, resulting in improved thermal characteristics. For comparison, our compounds demonstrated a significantly higher thermal stability than a well-defined spherosilicate with eight limonene fragments (Td_5_ = 109.6 °C) [49]. This difference is likely due to the greater number of limonene fragments in the spherosilicates, which may have decomposed first. The combination of the robust siloxane structure with the unique properties of terpene substituents has led to the development of new materials with improved thermal stability. This advancement expands the potential applications of both Janus ring siloxanes and terpenes, paving the way for the creation of high-performance materials in various fields such as biomedicine, coatings, and composites.

## 4. Conclusions

In this study, five novel Janus ring siloxanes with extended chains containing terpenes and terpenoids were successfully synthesized via a highly selective hydrosilylation reaction with a hydrosilyl-substituted Janus precursor. These materials, which were obtained under mild temperature conditions, were comprehensively characterized using multinuclear NMRs, MALDI-TOF mass spectroscopy, IR, and elemental analysis. The TGA results revealed the improved thermal stability of the synthesized Janus rings compared to the corresponding free essential oil components. Further investigations into other properties, such as the bioactivity or cytotoxicity of these compounds, are underway to explore the possibility of their use in medical and paramedical fields.

## Figures and Tables

**Table 1:** Comparative characteristics of compounds (**1**–**6**).

Compound	Isolated Yield (%)	29Si NMR, ppm	
		M-Unit	T-Unit
**1** [26]	80	−3.16	−78.08
**2**	75	11.06, 11.12	−79.87
**3**	77	11.04, 11.10	−79.87
**4**	65	10.66	−79.89
**5**	70	11.72	−79.36
**6**	53	10.79	−79.54

## Data Availability

All data and materials described in this work are available in this article or in Supplementary Materials.

## Supplementary Materials

The following supporting information can be downloaded at: https://www.mdpi.com/article/10.3390/ma17215348/s1: ^1^H, ^13^C, ^29^Si NMR, MALDI-TOF mass, and infrared spectra of compounds **2**–**6**; thermogravimetry/differential thermal analysis (TG/DTA) results for compounds **2**–**6**, and *S*-limonene, *R*-limonene, (-)-*β*-pinene, linalool, and eugenol. Figure S1: ^1^H NMR spectrum for compound **2**; Table S1: Thermal properties for compounds **2**-**6** and *S*-limonene, *R*-limonene, (-)-*β*-pinene, linalool, eugenol under N_2_; Figure S2: ^13^C NMR spectrum for compound **2**; Figure S3: ^29^Si NMR spectrum for compound **2**; Figure S4: ^1^H NMR spectrum for compound **3**; Figure S5: ^13^C NMR spectrum for compound **3**; Figure S6: ^29^Si NMR spectrum for compound **3**; Figure S7: ^1^H NMR spectrum for compound **4**; Figure S8: ^13^C NMR spectrum for compound **4**; Figure S9: ^29^Si NMR spectrum for compound **4**; Figure S10: ^1^H NMR spectrum for compound **5**; Figure S11: ^13^C NMR spectrum for compound **5**; Figure S12: ^29^Si NMR spectrum for compound **5**; Figure S13: ^1^H NMR spectrum for compound **6**; Figure S14: ^13^C NMR spectrum for compound **6**; Figure S15: ^29^Si NMR spectrum for compound **6**; Figure S16: MALDI-TOF MS spectrum for compound **2**; Figure S17: MALDI-TOF MS spectrum for compound **3**; Figure S18: MALDI-TOF MS spectrum for compound **4**; Figure S19: MALDI-TOF MS spectrum for compound **5**; Figure S20: MALDI-TOF MS spectrum for compound **6**; Figure S21: Infrared spectrum for compound **2**; Figure S22: Infrared spectrum for compound **3**; Figure S23: Infrared spectrum for compound **4**; Figure S24: Infrared spectrum for compound **5**; Figure S25: Infrared spectrum for compound **6**; Figure S26: Thermogravimetric graphs of essential oil components; Figure S27: Thermogravimetric graph of compound **2**; Figure S28: Thermogravimetric graph of compound **3**; Figure S29: Thermogravimetric graph of compound **4**; Figure S30: Thermogravimetric graph of compound **5**; Figure S31: Thermogravimetric graph of compound **6**; Figure S32: Thermogravimetric graph of ***S*-limonene**; Figure S33: Thermogravimetric graph of ***R*-limonene**; Figure S34: Thermogravimetric graph of **(-)-*β*-pinene**; Figure S35: Thermogravimetric graph of **linalool**; Figure S36: Thermogravimetric graph of **eugenol**.
