# Supplementary material for: Synthesis and Thermal Properties of Bio-Based Janus Ring Siloxanes Incorporating Terpenes and Terpenoids
Source: Materials (Basel). 2024 Oct 31;17(21):5348. doi: 10.3390/ma17215348 (PMC11547749; doi:10.3390/ma17215348)
Supplement: Supplementary file 1 [file materials-17-05348-s001.zip › materials-3032038-supplementary.pdf]

Synthesis and Thermal Properties of Biobased Janus Ring Siloxanes Incorporating Terpenes and Terpenoids

Niyaz Yagafarov[a], Jiaorong Kuang[a], Nobuhiro Takeda[a], Yujia Liu[a]*, Armelle Ouali[b]*, Masafumi Unno[a]

a. Gunma University, Department of Chemistry and Chemical Biology, 1-5-1 Tenjin-cho, Kiryu 376-8515, Japan
b. ICGM, Univ. Montpellier, CNRS, ENSCM, 1919 route de Mende, Montpellier 34293 Cedex 05, France

Supporting information

Table of contents **Pages**

1. ^{1}H, ^{13}C and ^{29}Si-NMR spectra for compounds **2-6** S2
2. MALDI-TOF MS spectra for compounds **2-6** S10
3. Infrared spectra for compounds **2-6** S13
4. Thermogravimetric analysis for compounds **2-6** and *S*-limonene, *R*-limonene, (-)-*β*-pinene, linalool, eugenol S15

1. ^{1}H, ^{13}C and ^{29}Si NMR spectra for compounds 2-6

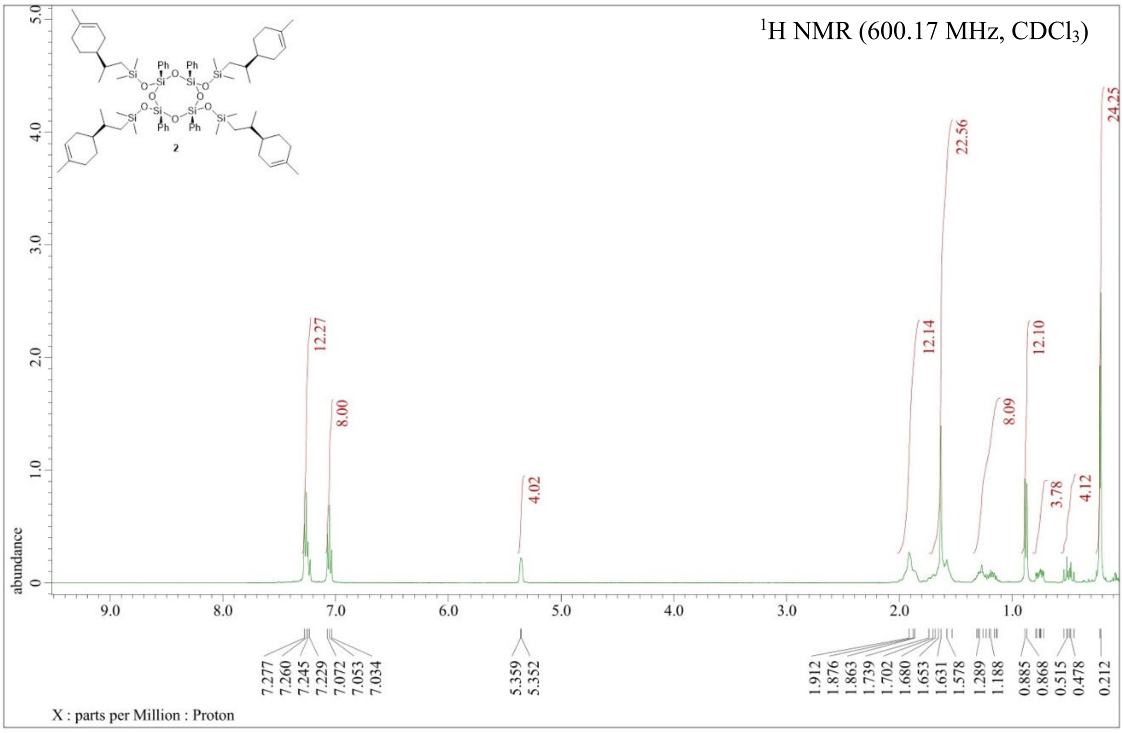

Figure S1: ^{1}H NMR spectrum for compound **2**

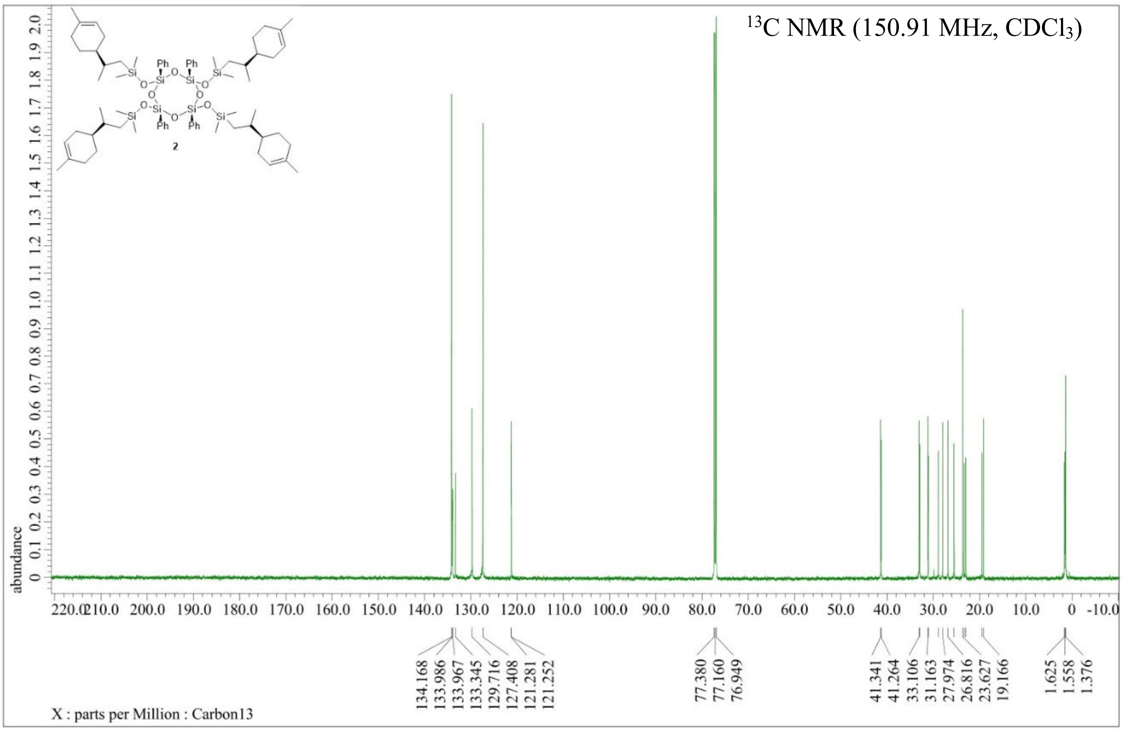

Figure S2: ^{13}C NMR spectrum for compound **2**

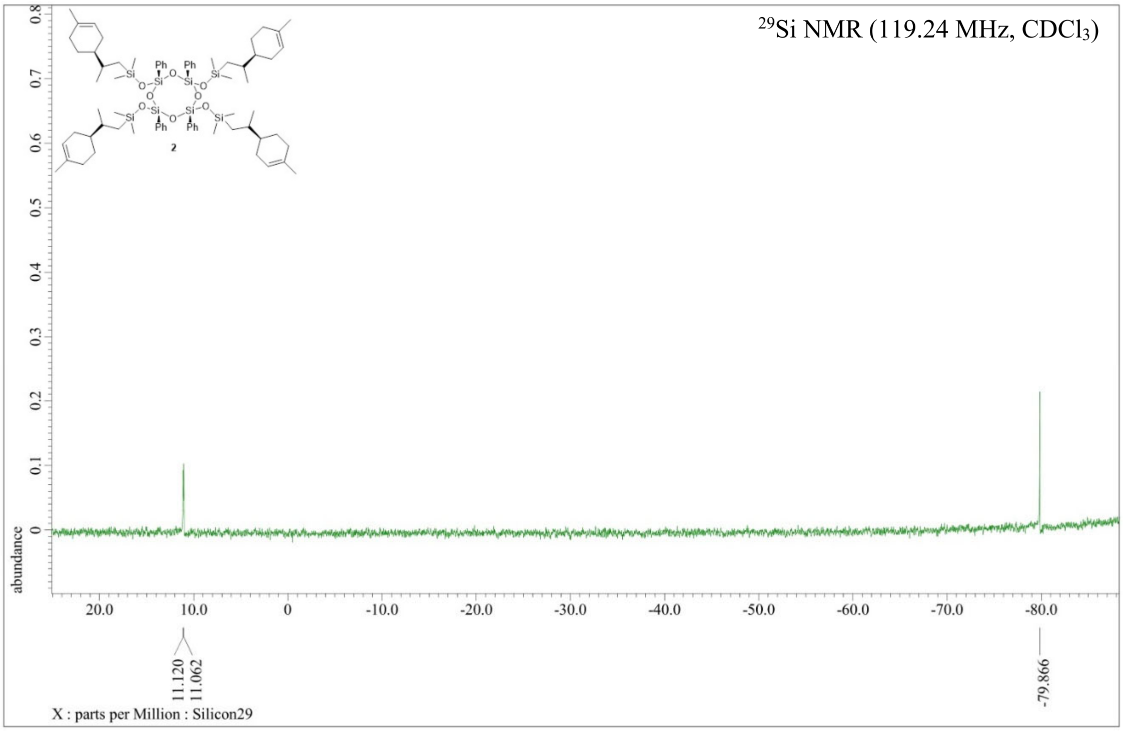

Figure S3: ^{29}Si NMR spectrum for compound **2**

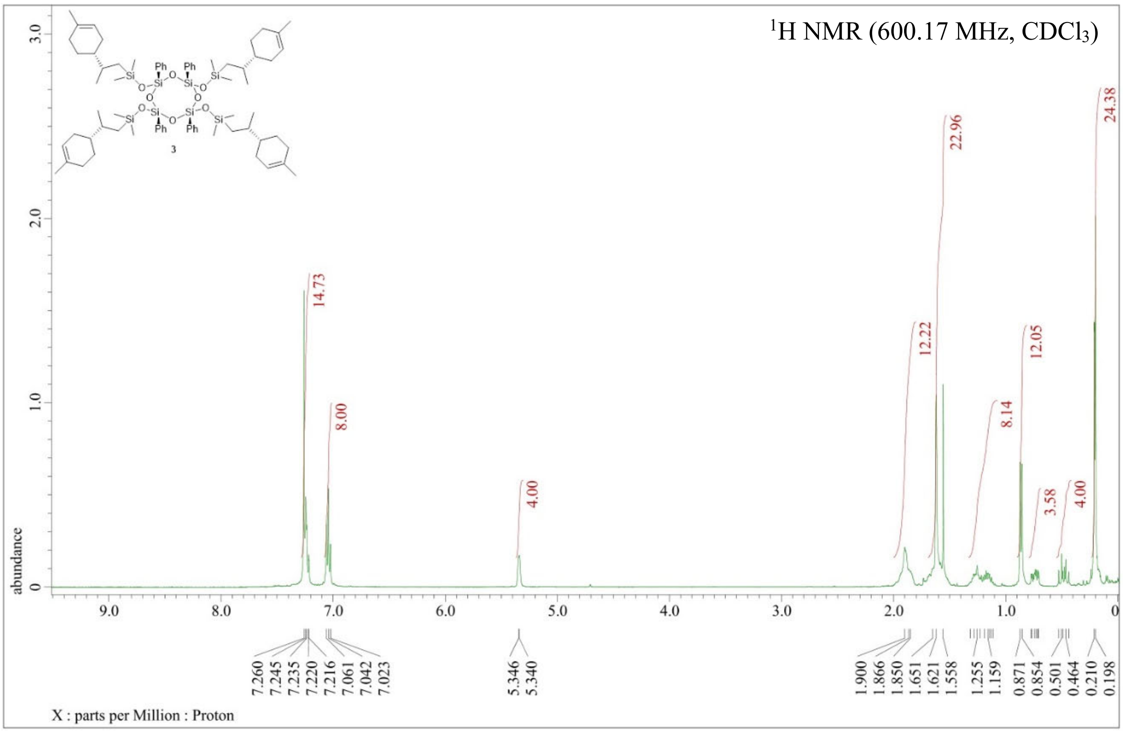

Figure S4: ^{1}H NMR spectrum for compound **3**

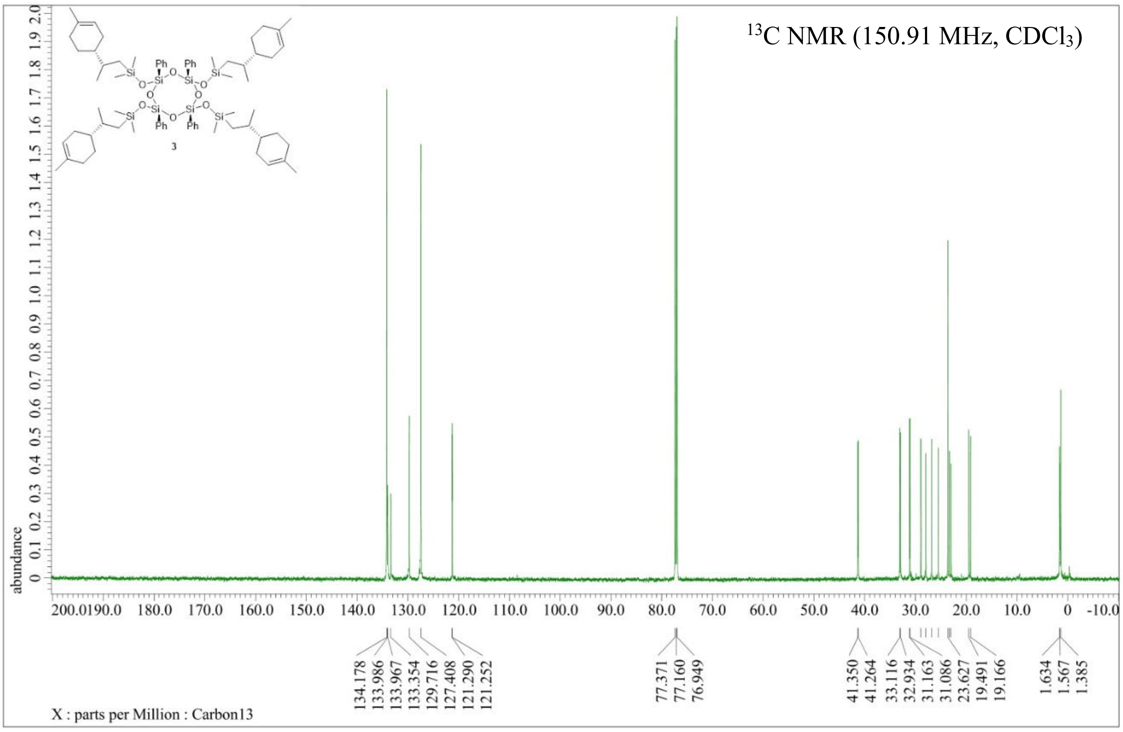

Figure S5: ^{13}C NMR spectrum for compound **3**

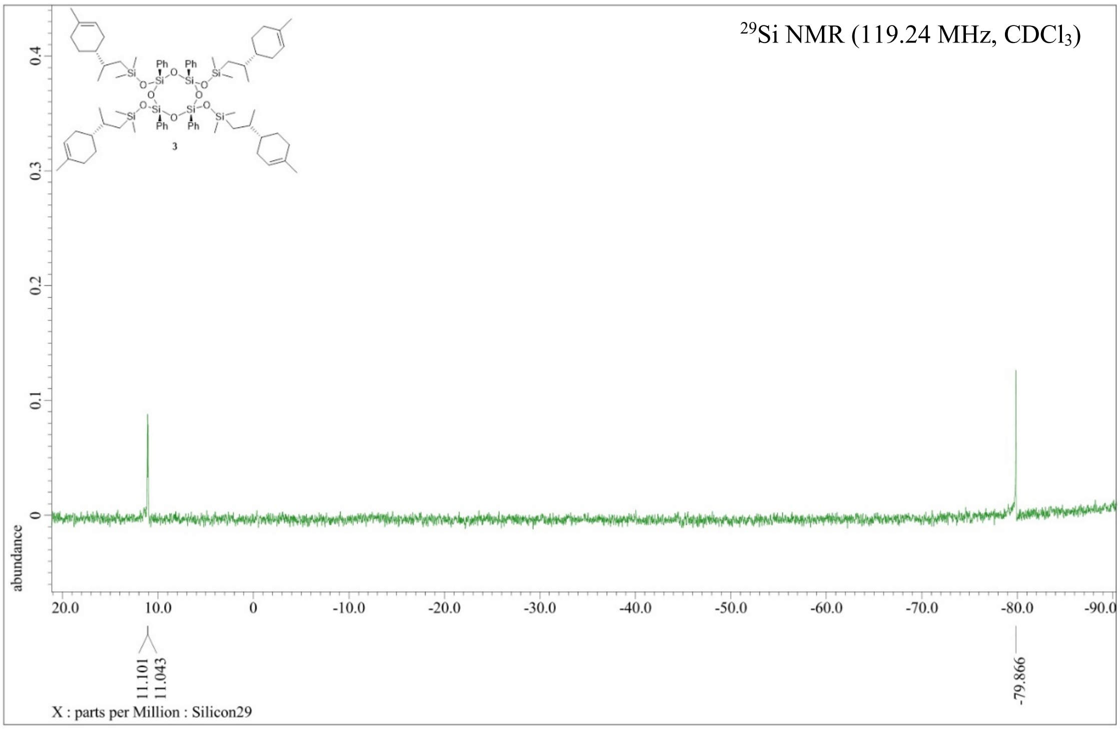

Figure S6: ^{29}Si NMR spectrum for compound **3**

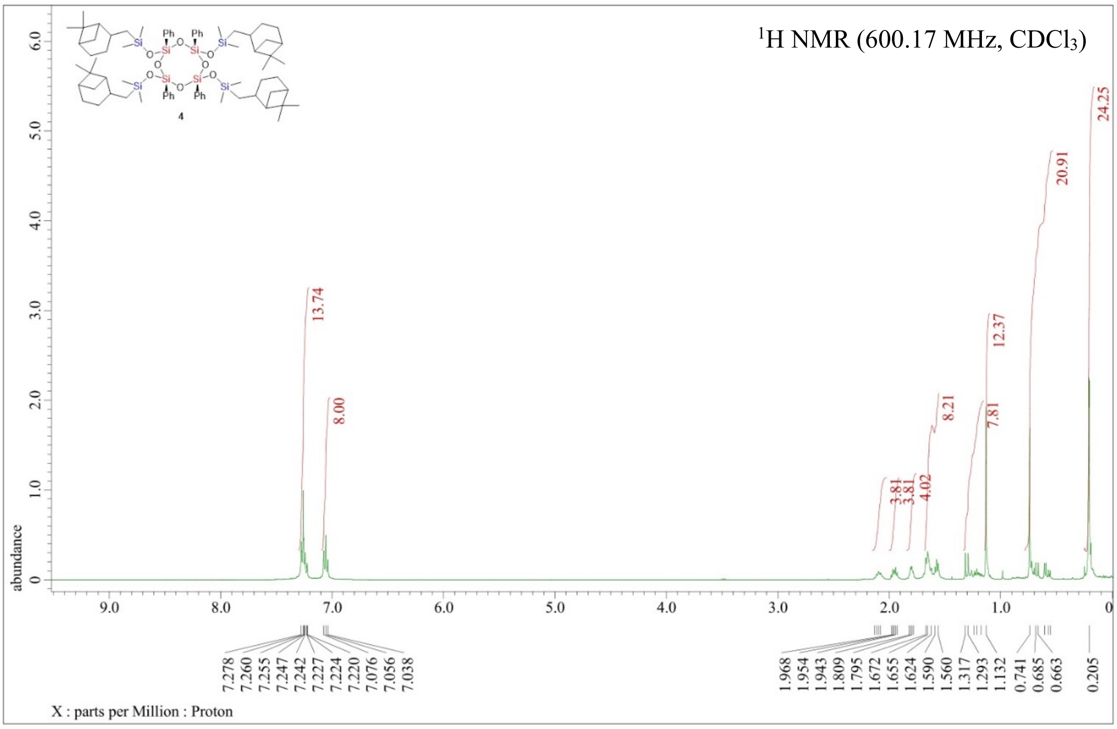

Figure S7: ^{1}H NMR spectrum for compound **4**

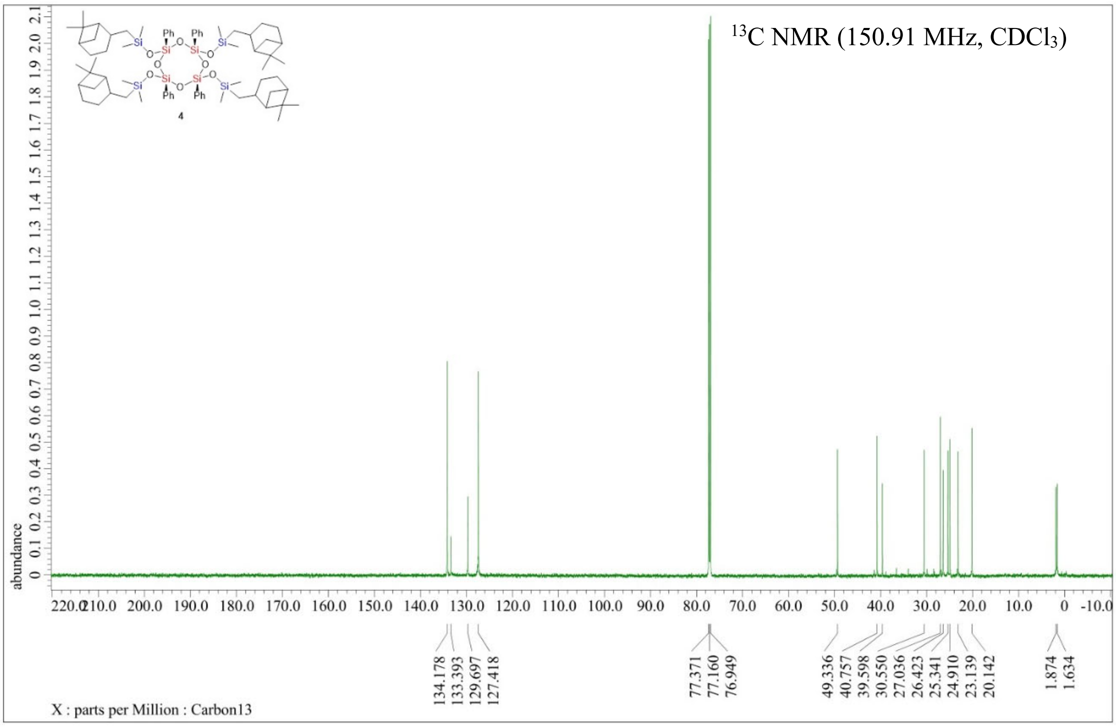

Figure S8: ^{13}C NMR spectrum for compound **4**

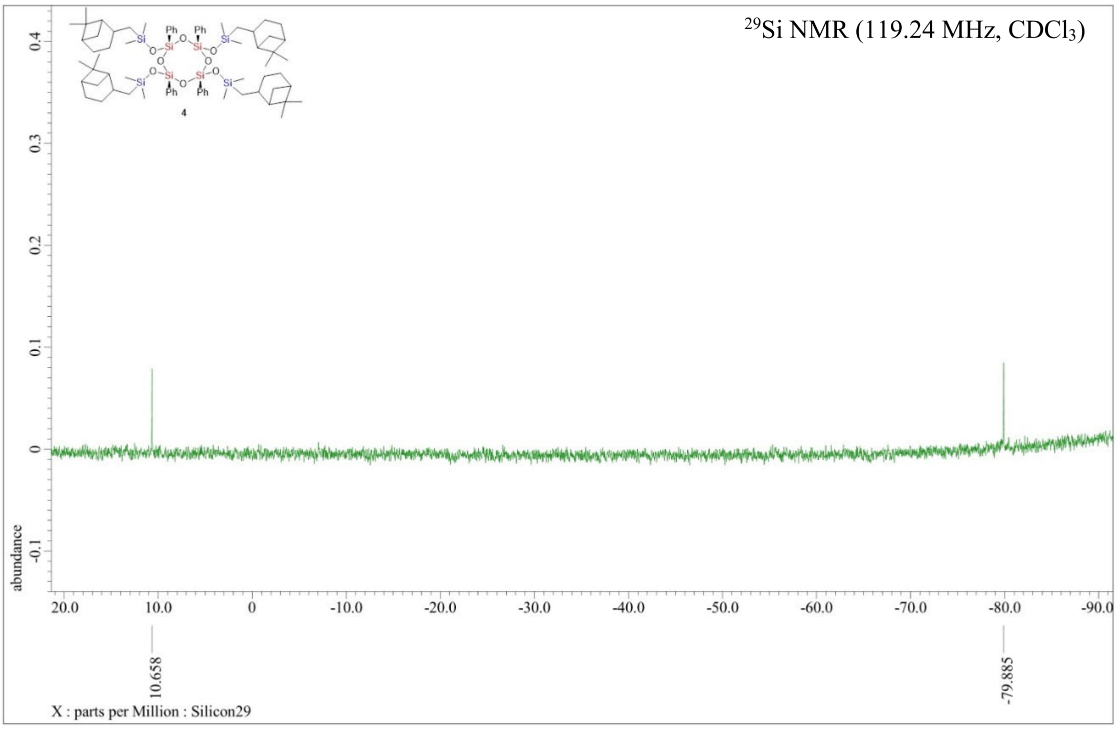

Figure S9: ^{29}Si NMR spectrum for compound **4**

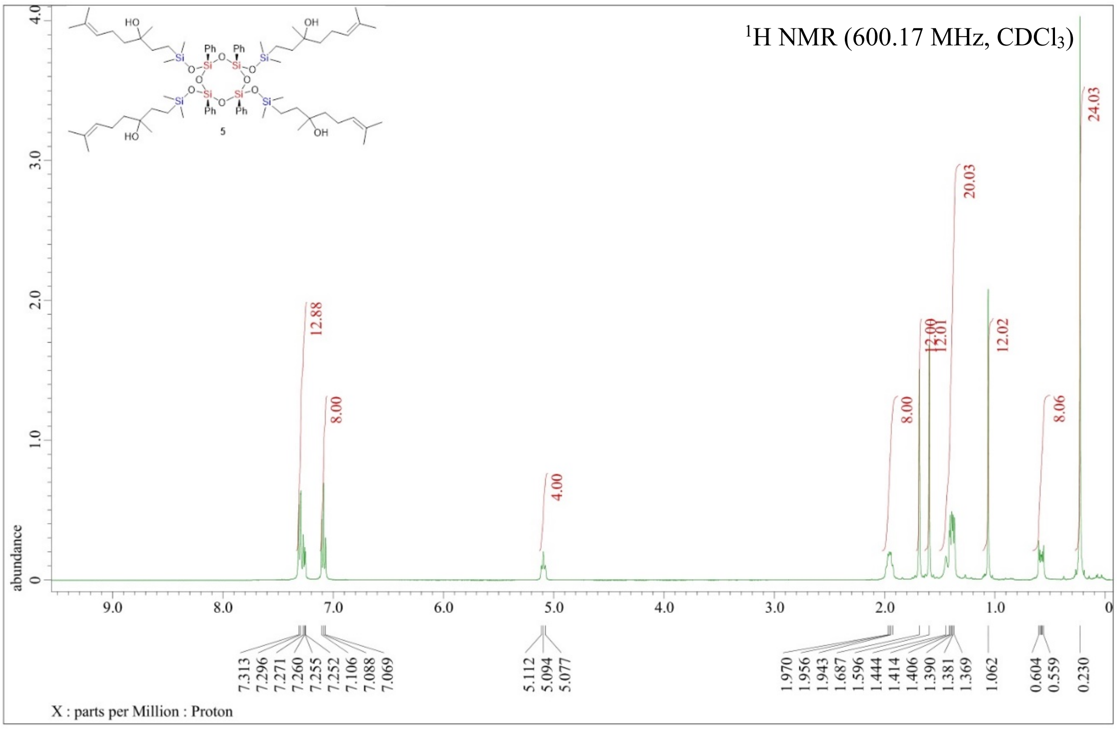

Figure S10: ^{1}H NMR spectrum for compound **5**

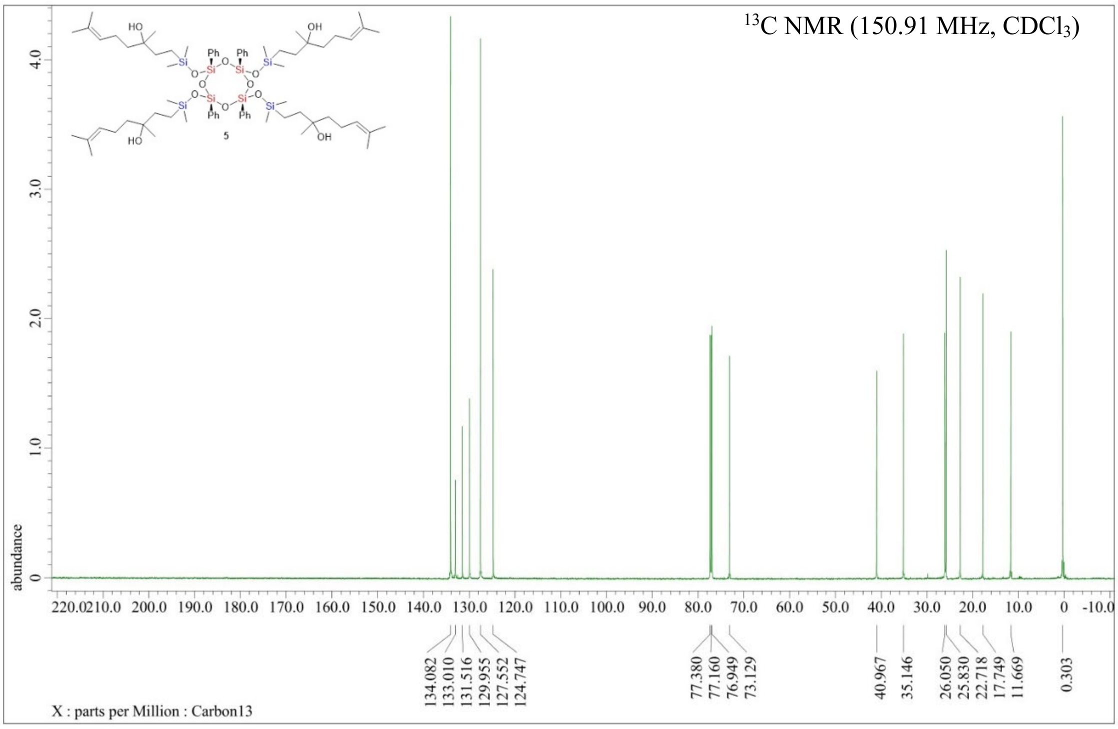

Figure S11: ^{13}C NMR spectrum for compound **5**

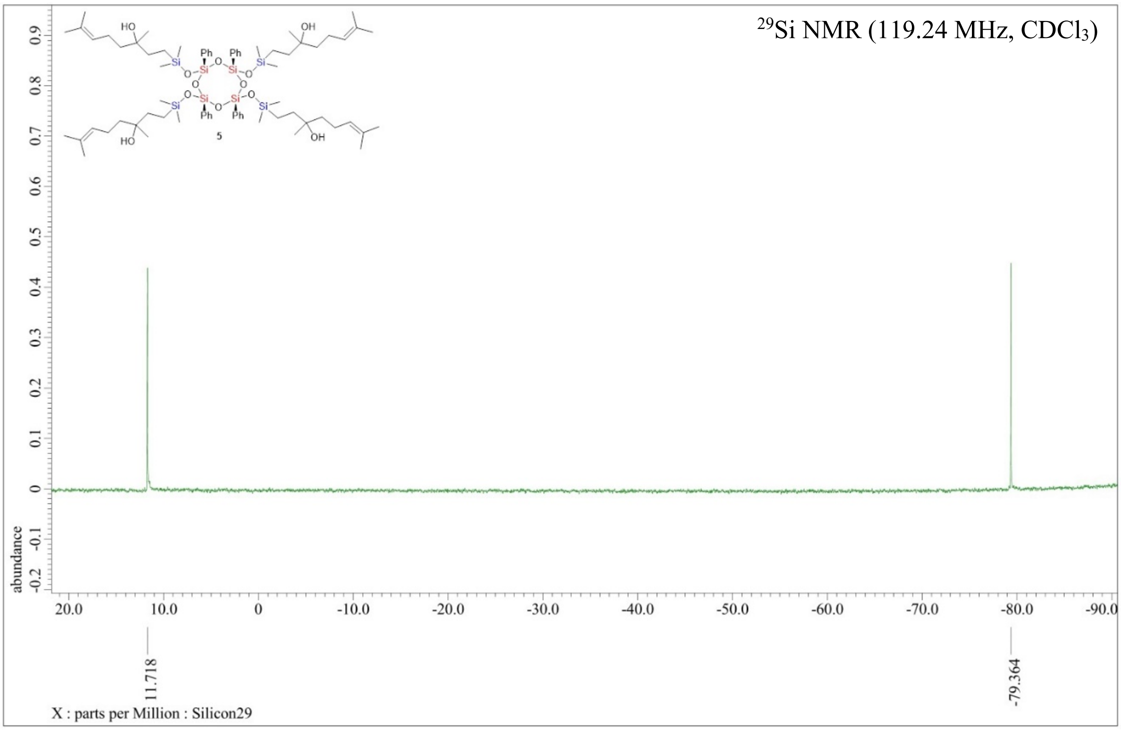

Figure S12: ^{29}Si NMR spectrum for compound **5**

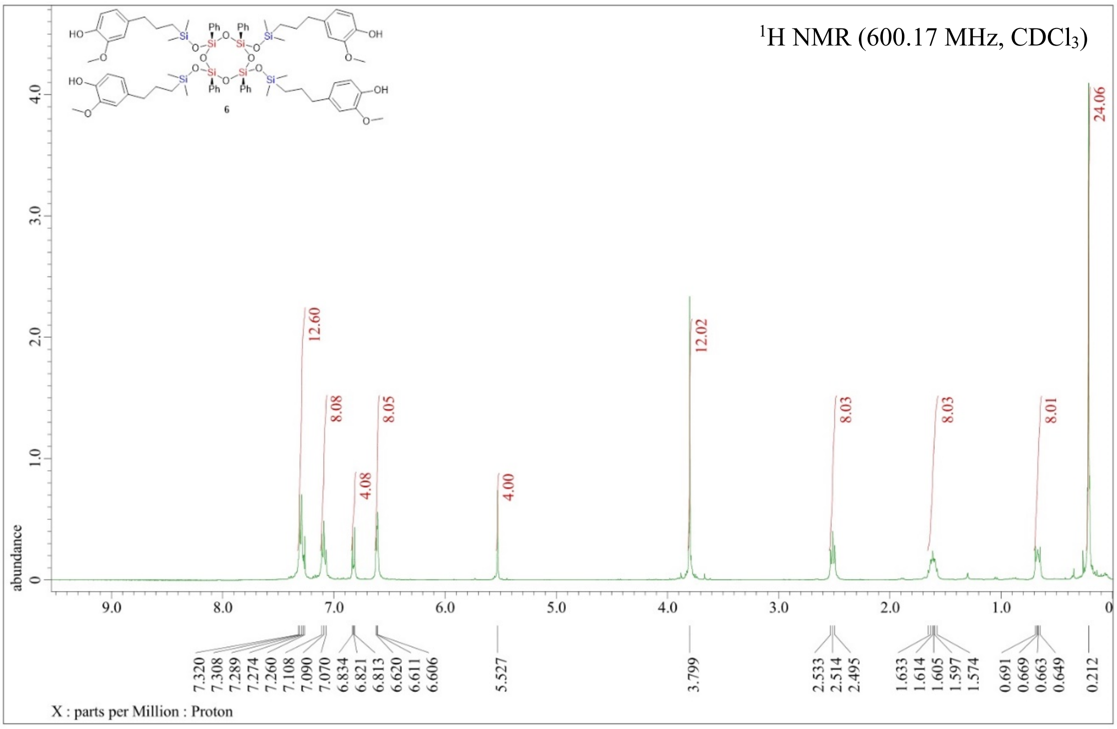

Figure S13: ^{1}H NMR spectrum for compound **6**

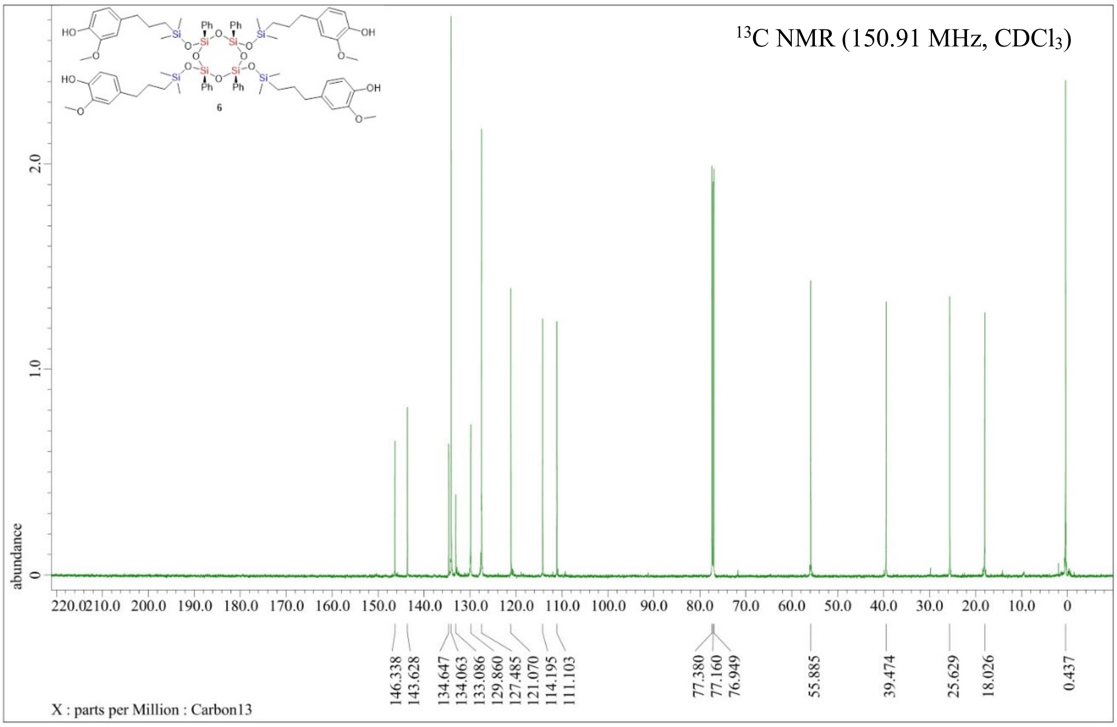

Figure S14: ^{13}C NMR spectrum for compound **6**

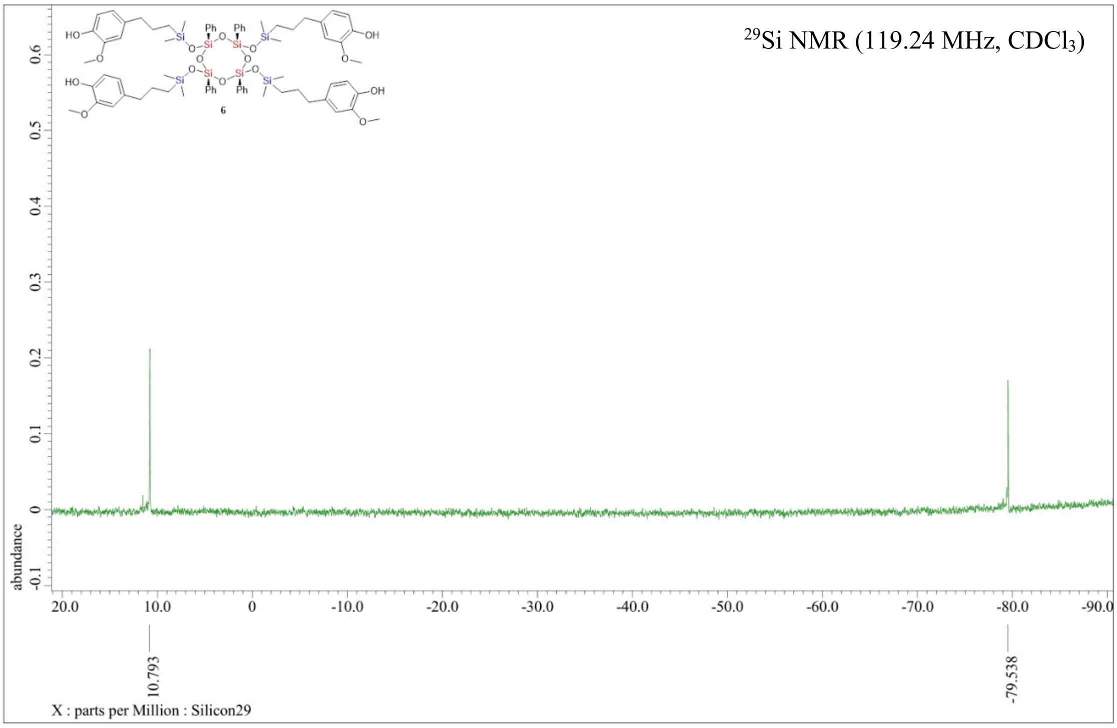

Figure S15: ^{29}Si NMR spectrum for compound **6**

4. MALDI-TOF MS spectra for compounds 2-6

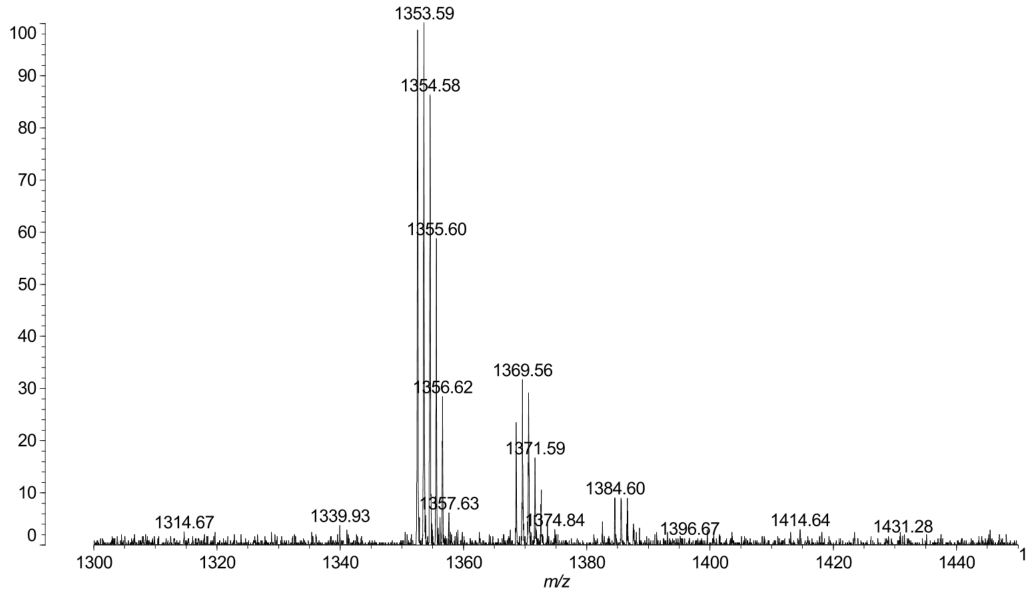

Figure S16: MALDI-TOF MS spectrum for compound **2**

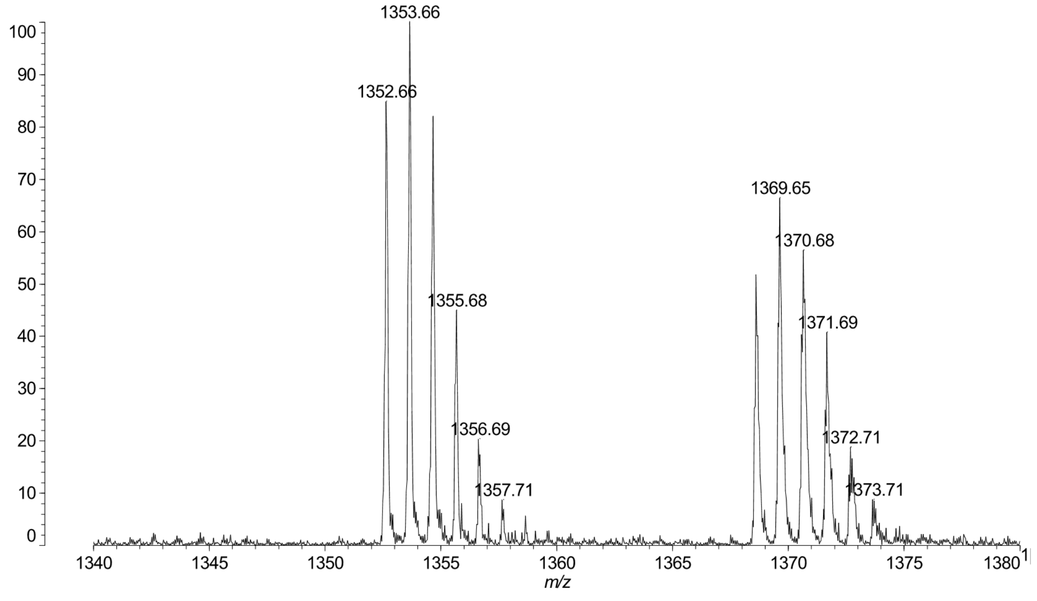

Figure S17: MALDI-TOF MS spectrum for compound **3**

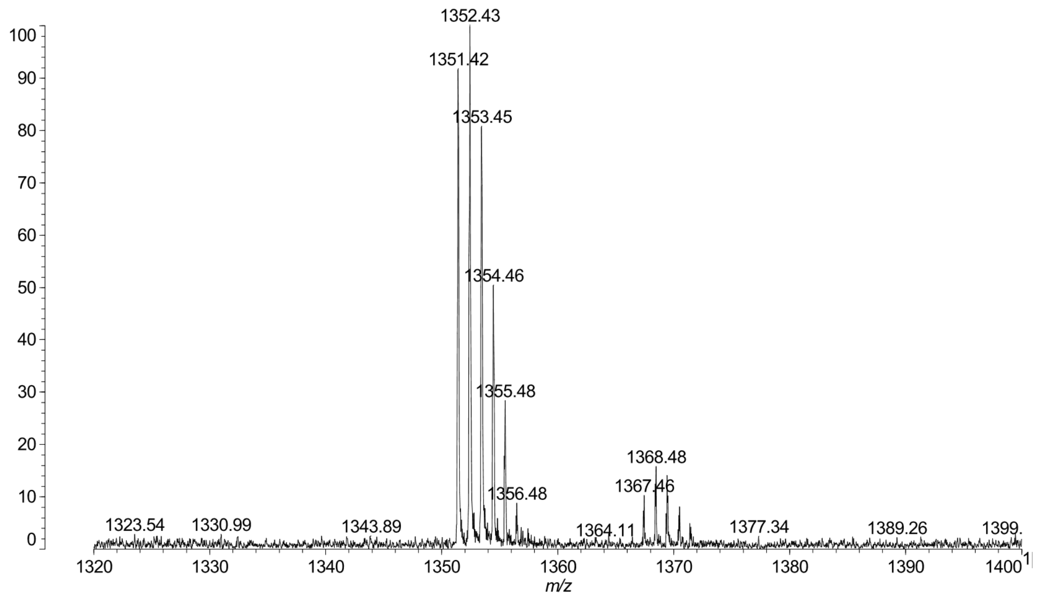

Figure S18: MALDI-TOF MS spectrum for compound **4**

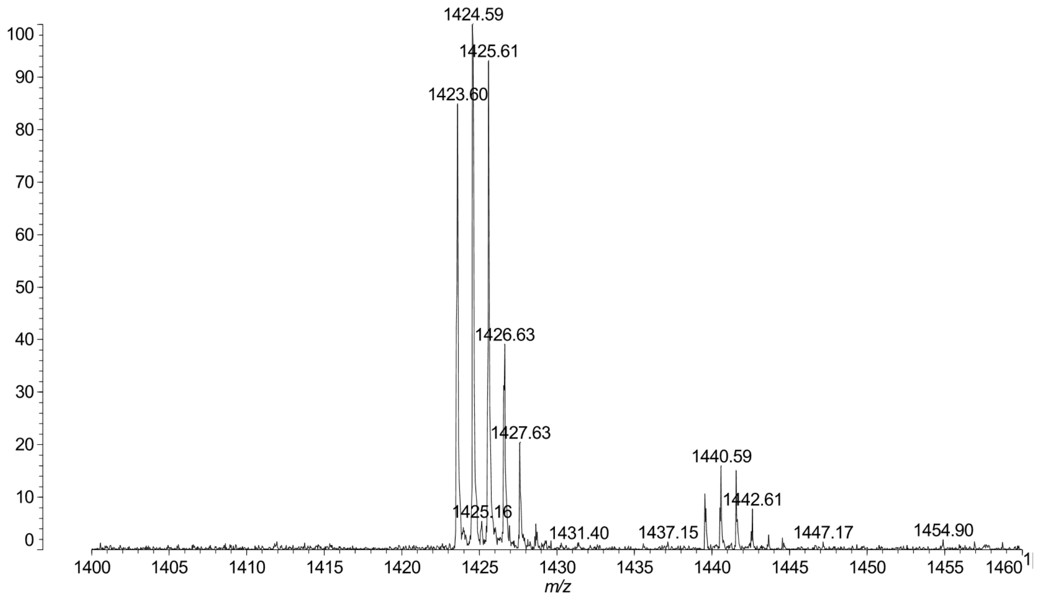

Figure S19: MALDI-TOF MS spectrum for compound **5**

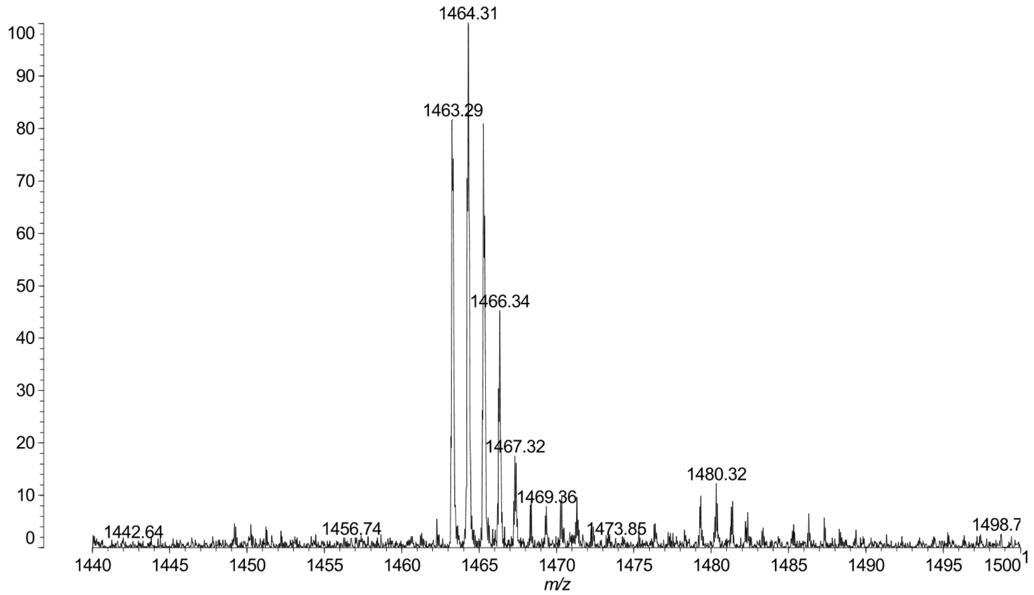

Figure S20: MALDI-TOF MS spectrum for compound **6**

5. Infrared spectra for compounds 2-6

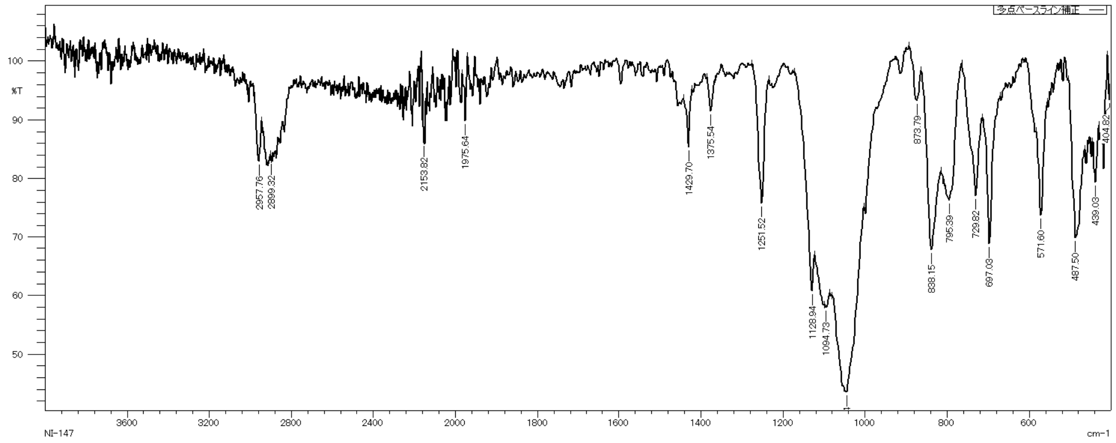

Figure S21: Infrared spectrum for compound **2**

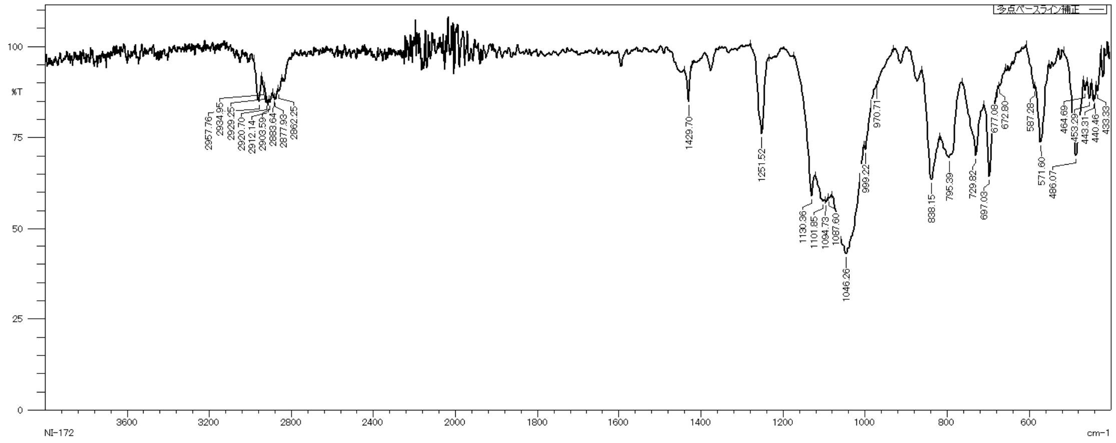

Figure S22: Infrared spectrum for compound **3**

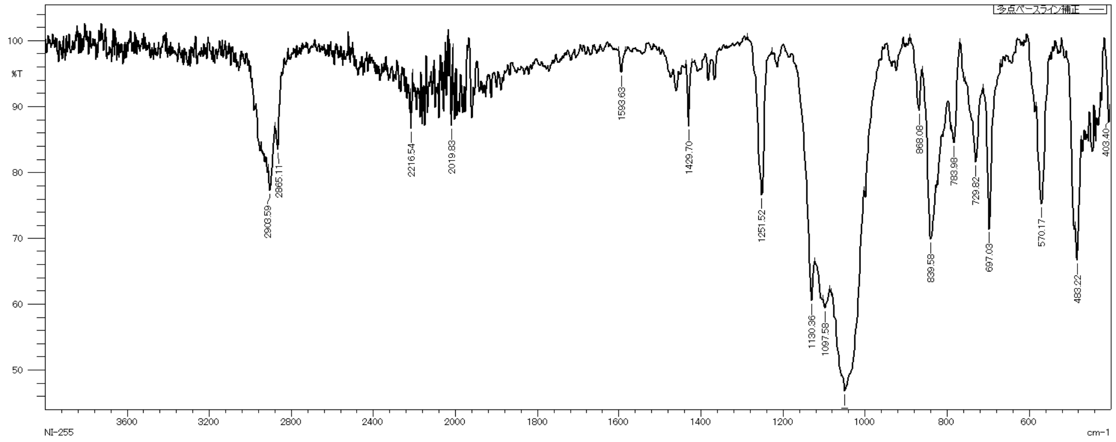

Figure S23: Infrared spectrum for compound **4**

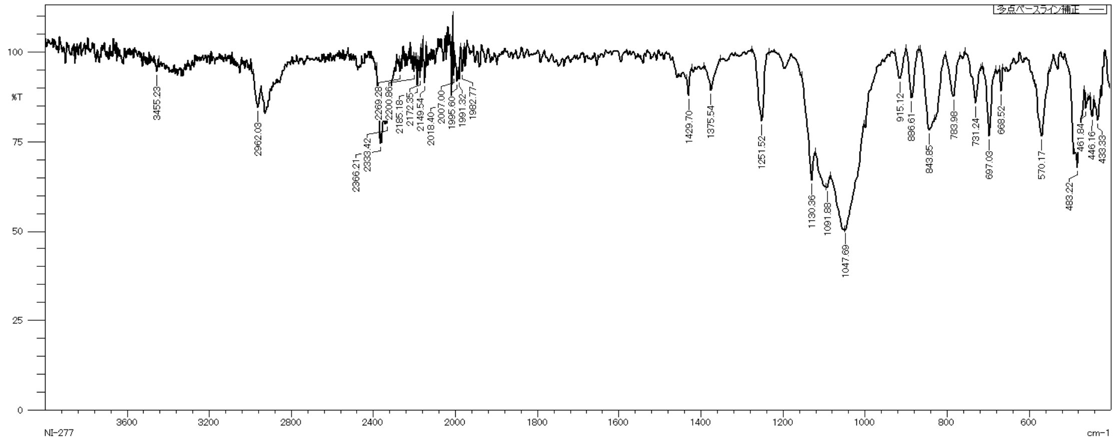

Figure S24: Infrared spectrum for compound **5**

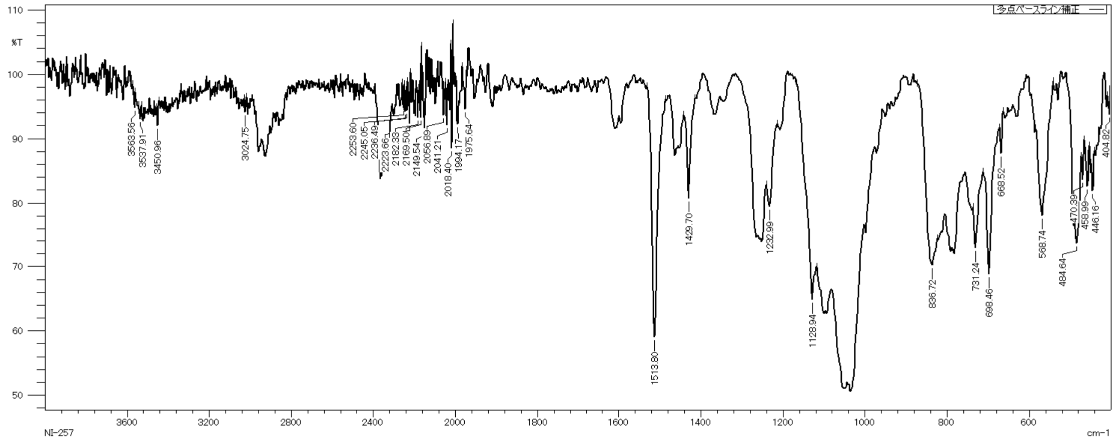

Figure S25: Infrared spectrum for compound **6**

6. Thermogravimetric analysis for compounds 2-6 and *S*-limonene, *R*-limonene, (-)-*β*-pinene, linalool, eugenol

Table S1. Thermal properties for compounds 2-6 and *S*-limonene, *R*-limonene, (-)-*β*-pinene, linalool, eugenol under N_2

Compounds	Si+O ratio (%)	Td_5 (°C)	Residue at 1000 °C (%)
**2**	26.51	322	6
**3**	26.51	354	17
**4**	26.51	296	2
**5**	29.71	239	26
**6**	33.33	256	30
***S*-limonene**	-	63	-
***R*-limonene**	-	60	-
**(-)-*β*-pinene**	-	51	-
**linalool**	-	80	-
**eugenol**	-	107	-

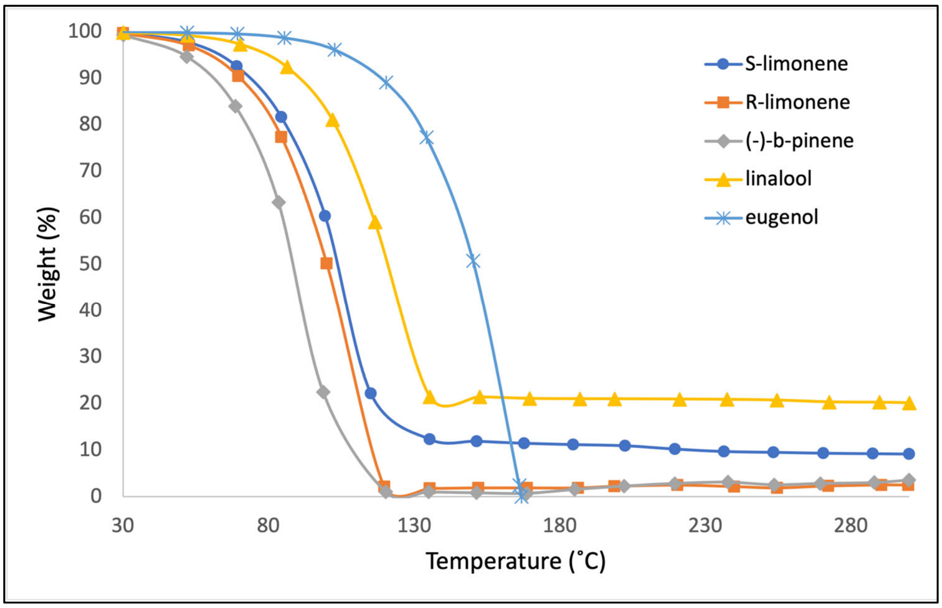

Figure S26: Thermogravimetric graphs of essential oil components

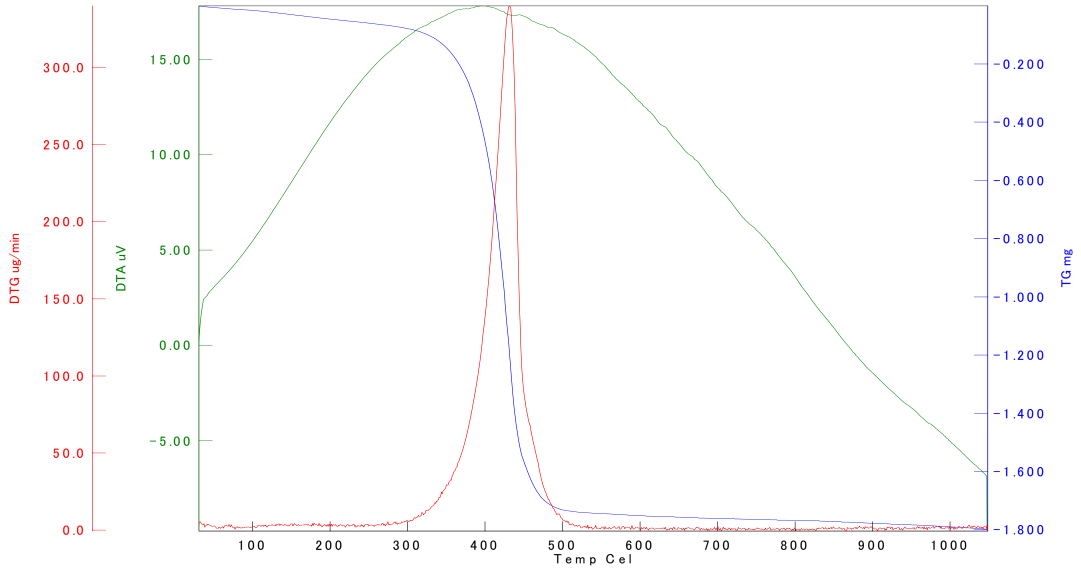

Figure S27: Thermogravimetric graph of compound **2**

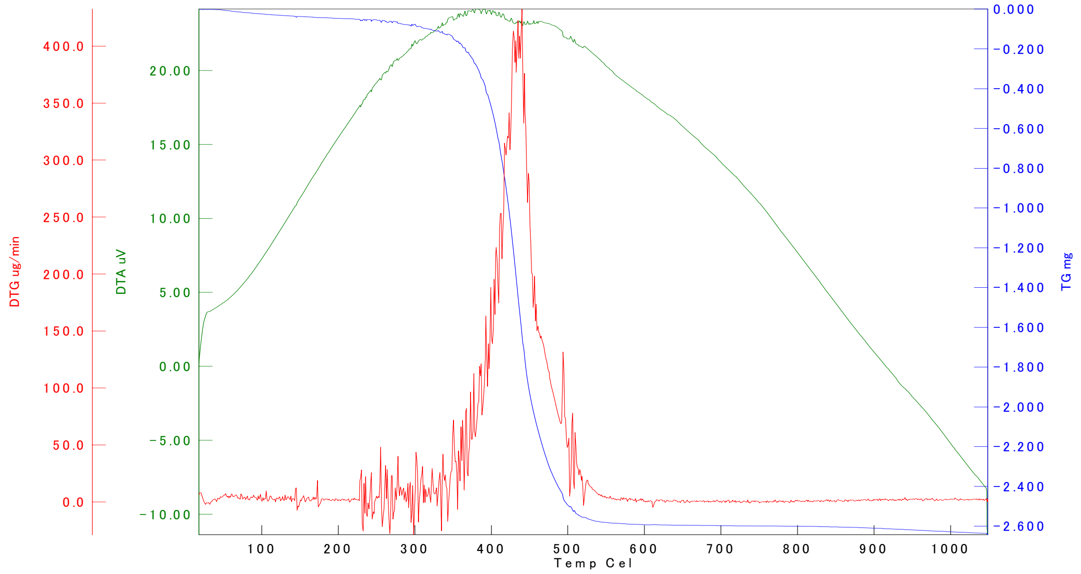

Figure S28: Thermogravimetric graph of compound **3**

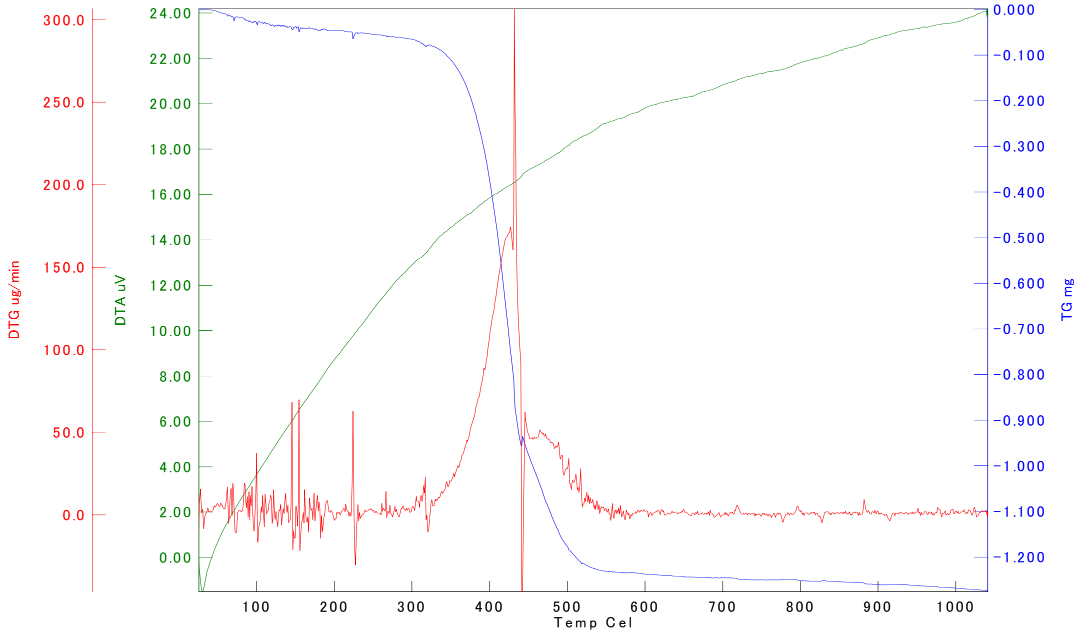

Figure S29: Thermogravimetric graph of compound **4**

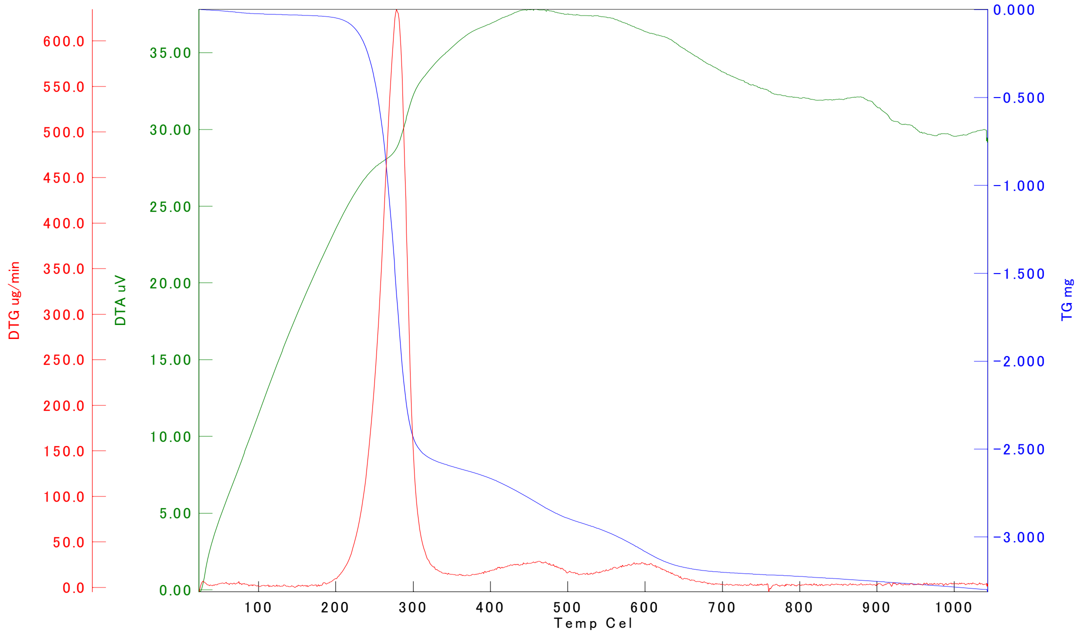

Figure S30: Thermogravimetric graph of compound **5**

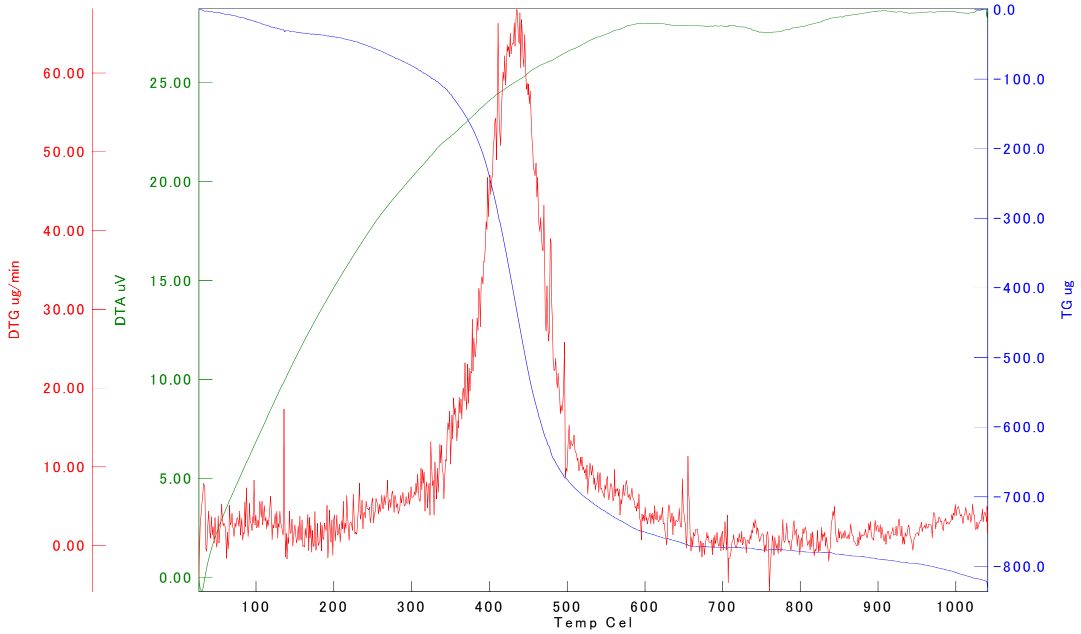

Figure S31: Thermogravimetric graph of compound **6**

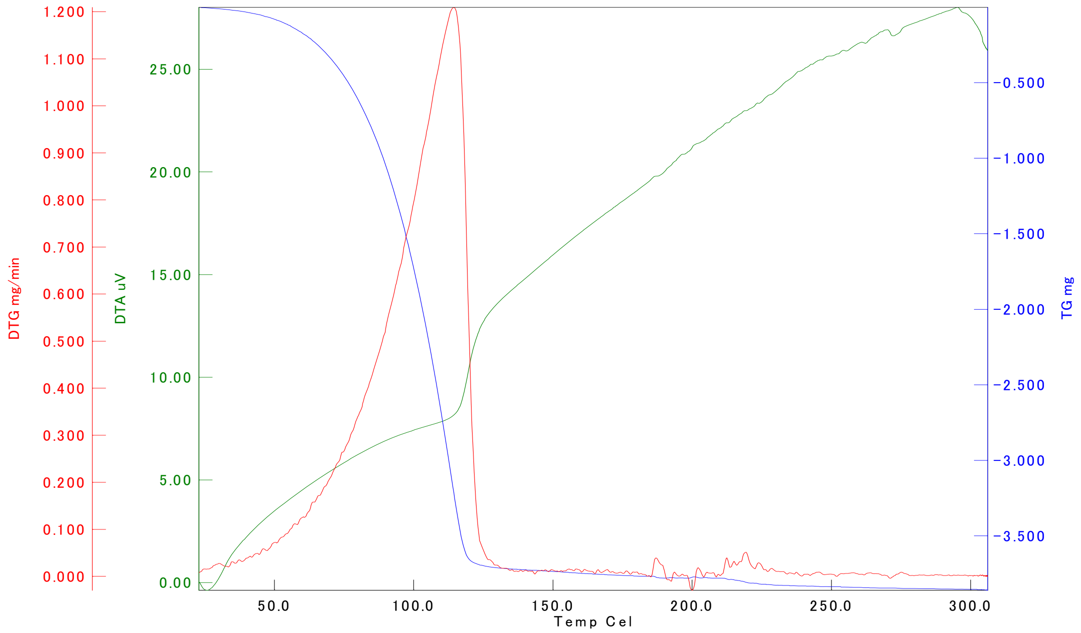

Figure S32: Thermogravimetric graph of ***S*-limonene**

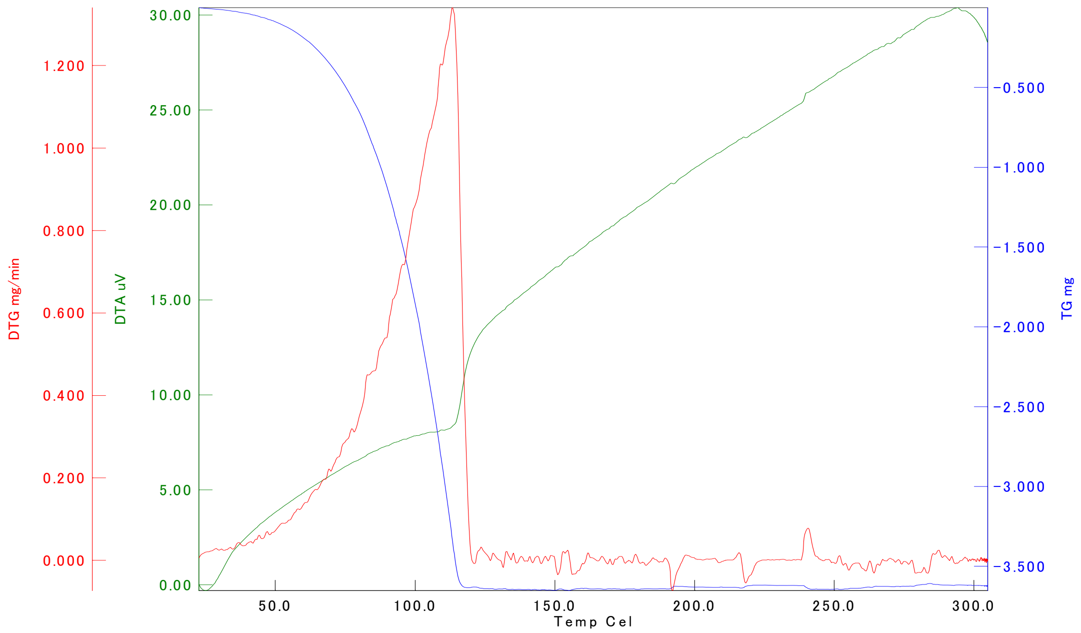

Figure S33: Thermogravimetric graph of ***R*-limonene**

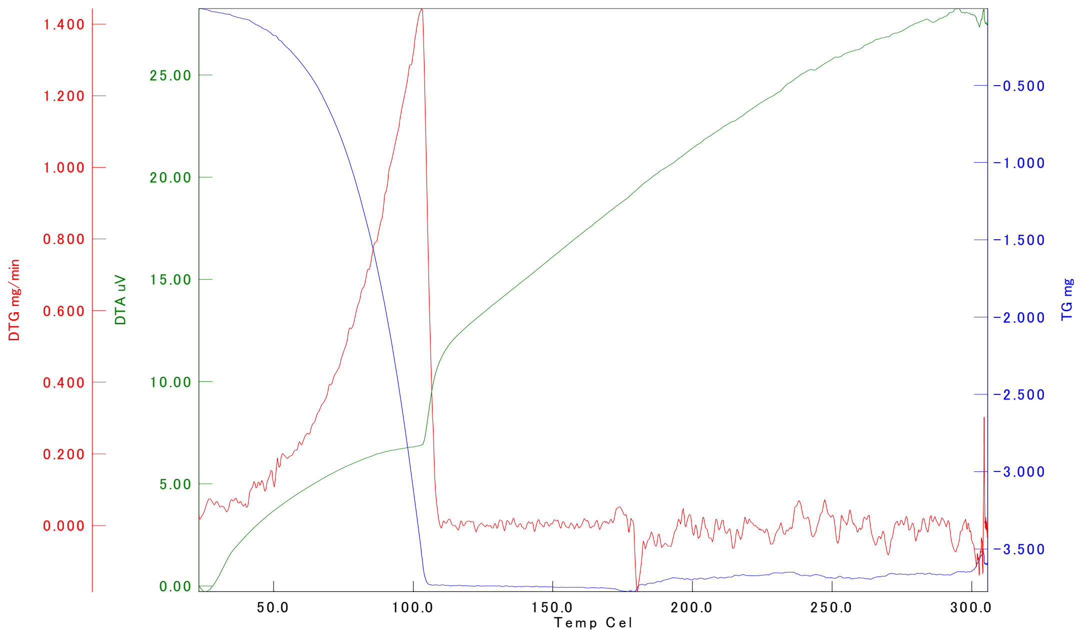

Figure S34: Thermogravimetric graph of **(-)-*β*-pinene**

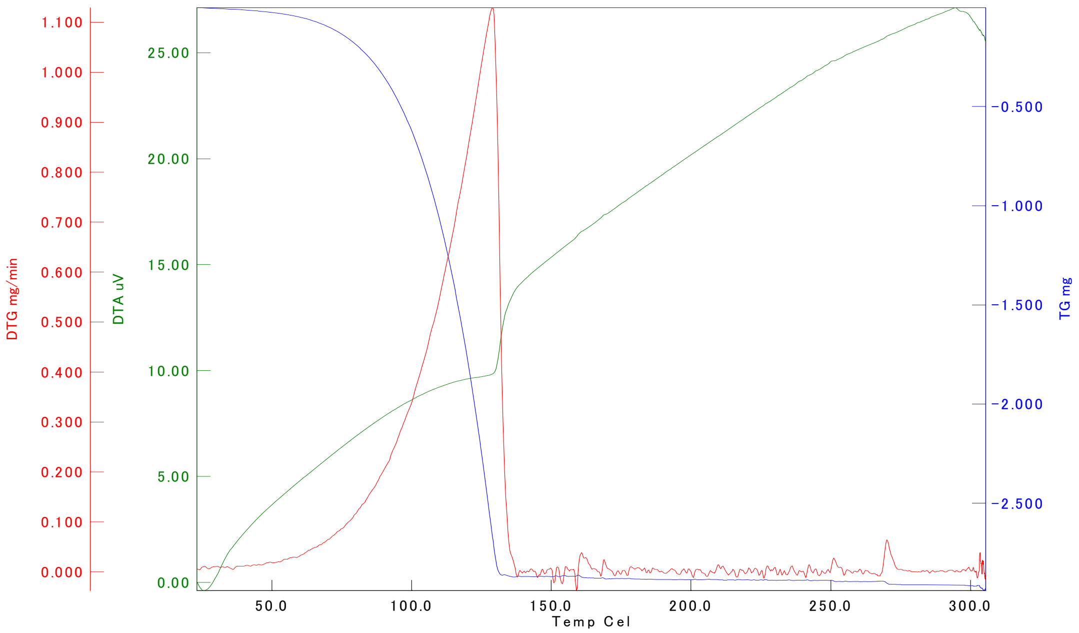

Figure S35: Thermogravimetric graph of **linalool**

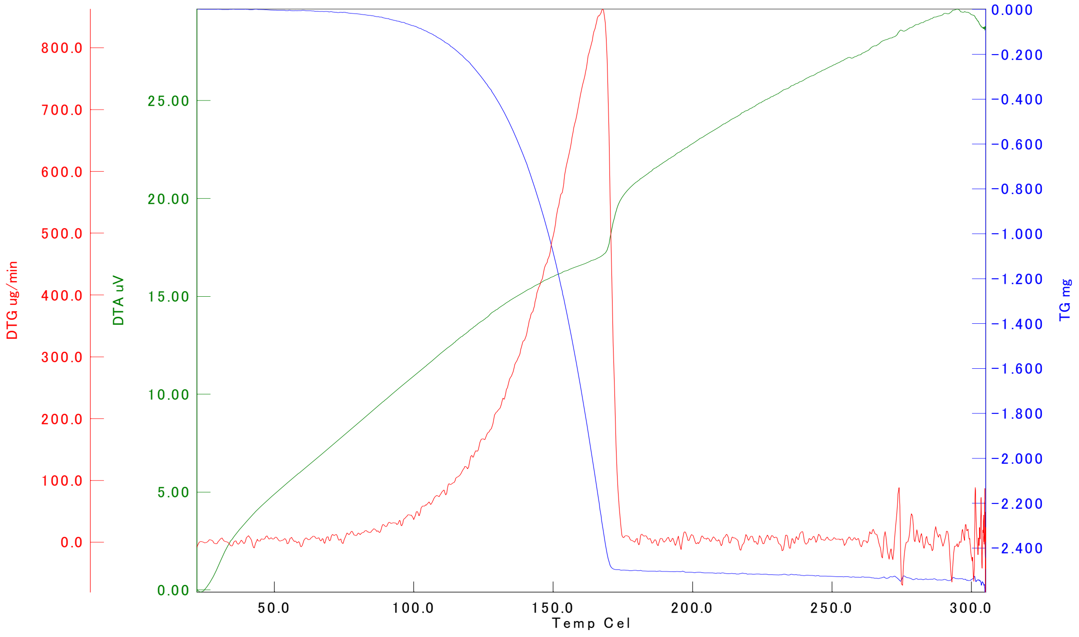

Figure S36: Thermogravimetric graph of **eugenol**
